# Immobilization of Enzyme–Polymer Hybrids and Nanozymes Through Electrostatic Interactions: Toward Multicatalytic Microreactors with Controlled Nanoarchitecture

**DOI:** 10.1002/smsc.202500167

**Published:** 2025-06-10

**Authors:** Aitor Ontoria, Irene Alonso‐Sampedro, Yixuan Yan, Ayşe Latif, Ben F. Spencer, Aitor Larrañaga, Ana Beloqui, Christos Tapeinos

**Affiliations:** ^1^ POLYMAT and Department of Applied Chemistry University of the Basque Country (UPV/EHU) Donostia‐San Sebastián 20018 Spain; ^2^ Department of Mining‐Metallurgy Engineering and Materials Science POLYMAT Bilbao School of Engineering University of the Basque Country (UPV/EHU) Plaza Torres Quevedo 1 Bilbao 48013 Spain; ^3^ Division of Pharmacy and Optometry Faculty of Biology, Medicine and Health University of Manchester Stopford Building, Oxford Road Manchester M13 9PT UK; ^4^ Department of Materials and Henry Royce Institute The University of Manchester Oxford Road Manchester M13 9PL UK; ^5^ IKERBASQUE Basque Foundation for Science Bilbao 48009 Spain

**Keywords:** CaCO_3_ templated assemblies, compartmentalized enzymatic cascades, layer‐by‐layer, Mn_3_O_4_ nanozymes, multilayer microreactors

## Abstract

The optimal allocation of catalysts and their precise compartmentalization are vital to ensure efficient cascade reactions. The layer‐by‐layer approach offers the possibility of assembling various building blocks onto templates of different sizes and shapes, thus representing a powerful tool for fabricating multicatalytic reactors with controlled nanoarchitecture. However, this process usually relies on electrostatic interactions between building blocks, which means a limitation when working with natural enzymes. Accordingly, both the loading capacity and control over membrane architecture are compromised by the inherent surface charge of the enzymes. Here, this study introduces a modular strategy to assemble engineered enzyme‐polymer hybrids and inorganic nanozymes onto colloidal templates, giving rise to multicatalytic reactors. The surface charge of the engineered enzyme–polymer hybrids can be finely tuned, allowing their à‐la‐carte assembly into multilayer membranes. Following this approach, the distance between catalytic units and their arrangement on colloidal templates at the nano‐ and micrometer scale can be precisely controlled, resulting in optimized configurations with enhanced cascade efficiency. The synthesized multicatalytic reactors can reduce the metabolic activity of human pancreatic stellate cells, confirming their functional activity in biological microenvironments and highlighting their potential for biomedical applications.

## Graphical abstract

A modular hybrid membrane is constructed via a layer‐by‐layer strategy, integrating glucose oxidase/horseradish peroxidase or glucose oxidase/nanozyme‐loaded microreactors within alternating polyelectrolyte layers on a calcium carbonate core. This hierarchical design enables precise spatial confinement of catalytic units, facilitating efficient cascade reactions. These microrreactors have potential biomedical applications, particularly in oxidative stress modulation and disruption of tumor‐stroma interactions.

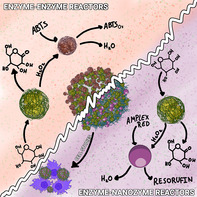

## 1 Introduction

Enzymes play a crucial role in mediating most (bio)chemical reactions involved in the biological processes of living organisms. Their compartmentalization enables the coexistence of competing enzymatic reactions within cells or their membranes, regulating resistance to toxic enzymatic intermediates and coordinating the biochemical cascades.^[^ ^1^, ^2^ ^]^ Artificial cells or protocells are supramolecular systems that mimic living cells’ behavior, function, and structure.^[^ ^3^ ^]^ They represent responsive multifunctional systems that exhibit controlled compartmentalization of enzymes working concomitantly. One strategy to design artificial cells with compartmentalized architecture relies on biologically relevant molecules such as DNA^[^ ^4^, ^5^ ^]^ or lipids.^[^ ^6^ ^]^ Alternatively, polymer architectures, ranging from polymersomes (i.e., polymer vesicles)^[^ ^7^ ^]^ to macroscopic gels, have also been explored. Thanks to their tunable properties and architecture, polymers offer many possibilities for compartmentalization.^[^ ^8^ ^]^ However, these architectures still face important challenges. For example, polymersomes exhibit mass transfer limitations across polymeric membranes and low entrapment of catalysts,^[^ ^9^, ^10^, ^11^ ^]^ negatively impacting the final product's activity.^[^ ^11^ ^]^ Therefore, developing new methods for strategically localizing demanded enzymes within assembled architectures is essential to protect their integrity and enable the effective performance of complex processes such as enzymatic cascade reactions.

Numerous research studies have focused on enzymatic cascade reactions in artificially assembled platforms, particularly one‐pot concurrent reactions.^[^ ^12^, ^13^ ^]^ In nature, the diffusion of substrates and intermediate products between enzymes and their environment ensures the efficiency of these processes. Therefore, the optimal allocation of such enzymes is vital for successfully orchestrating cascade reactions that drive biological processes. In this context, compartmentalized nano‐ and microparticles have emerged as excellent candidates to precisely tune the localization of enzymes into compartments with confined volumes. In traditional materials, enzymes are co‐immobilized uniformly on the surface (e.g., polymeric matrices or inorganic nanoparticles).^[^ ^14^ ^]^ In contrast, the controlled allocation of the enzymes gives rise to coordinated reactors. These reactors drive the process toward the desired equilibrium reaction. Additionally, they help regulate substrate channeling and catalytic activity, overcoming diffusion limitations and facilitating the transport of intermediates to their target enzyme.^[^ ^15^ ^]^ As a result, benefits such as lower operating costs, reduced separation time, and improved waste treatment are expected. However, the success of the close‐localization confined volumes in the same environment relies on such enzymes’ functional and structural compatibility. Thus, the catalytic performance of enzymes with distinct operational conditions, such as pH and temperature, can be compromised in cascade reactions.^[^ ^16^ ^]^ To address this challenge, several investigations have placed the combination of enzymes with robust nanozymes in the spotlight.

While controversial,^[^ ^17^ ^]^ nanozymes are generally considered nanomaterials with enzyme‐like activity. In 2021, nanozymes were defined as “nanomaterials that catalyze the conversion of enzyme substrates to products and follow enzymatic kinetics (e.g., Michaelis–Menten) under physiologically relevant conditions.” However, the molecular mechanisms of the reactions can be different between nanozymes and their natural counterparts.^[^ ^18^ ^]^ Their ability to perform under physiologically relevant conditions makes them well‐suited to complement enzymes for chemoenzymatic cascade reactions. Among the reported nanozymes, Mn_3_O_4_ nanoparticles show promising characteristics that allow them to collaborate with enzymes. These Mn_3_O_4_ nanozymes exhibit various catalytic activities like catalase,^[^ ^19^, ^20^ ^]^ oxidase,^[^ ^21^ ^]^ and peroxidase,^[^ ^22^ ^]^ which makes them ideal candidates for enzyme–nanozyme systems. However, combining them into practical platforms requires a suitable strategy, which has not yet been thoroughly explored.

In this work, we developed a simple and versatile strategy to tailor the localization of enzymes and nanozymes within assembled structures. We hypothesized that this would facilitate the assembly of multicatalytic systems with potential theragnostic applications in biomedical settings. We have focused our research on engineering multilayered membranes deposited on the surface of a CaCO_3_ inorganic core employed as a proof‐of‐concept colloidal template. The strategy is based on building the membrane using the layer‐by‐layer (LbL) approach, in which both polyelectrolytes and catalytically active building blocks (i.e., enzyme–polymer hybrids and nanozymes) are assembled on demand.^[^ ^23^ ^]^ LbL has been widely applied in the biomedical field as a delivery vehicle for drugs,^[^ ^24^ ^]^ genes,^[^ ^25^ ^]^ and proteins.^[^ ^26^ ^]^ However, to our knowledge, current strategies for protein immobilization within layers rely on covalent attachment or affinity binding.

**Scheme 1:**
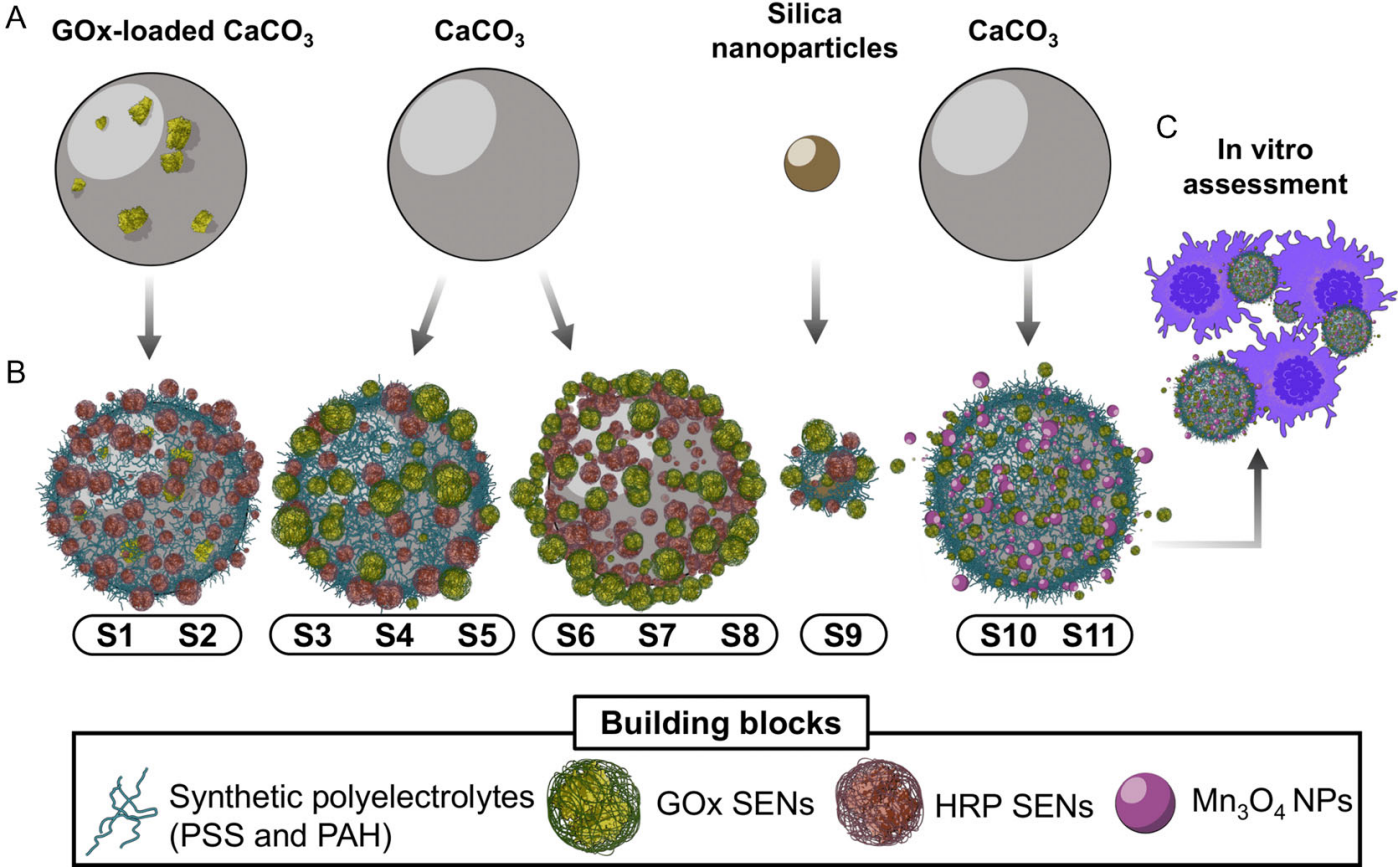
Depiction of the strategies and the composition of the materials utilized in this work to build the engineered (hybrid) membranes. A) Three distinct templates were used in the fabrication of the samples: GOx‐loaded CaCO_3_, bare CaCO_3_ (unloaded), and mesoporous silica nanoparticles (MSN). B) Following the application of the LbL technique, various building blocks were assembled onto the template surfaces, resulting in a small library of catalytic membranes capable of executing the selected cascade reaction, either through the collaborative action of GOx and HRP enzymes or the combination of the GOx enzyme and Mn_3_O_4_ nanoparticles. C) The S11 sample was further utilized for preliminary in vitro studies.

However, these methods often yield low immobilization efficiency and require chemical modification of polyelectrolytes, which can lead to stability issues.^[^ ^27^ ^]^ In contrast, the immobilization of proteins through electrostatic interactions, which could help broaden the versatility of this approach, has not been adequately investigated. Herein, enzyme‐polymer hybrids are proposed to overcome the current limitations to the efficient immobilization of enzymes via the LbL approach. In particular, single‐enzyme nanogels (SENs) are proposed as building blocks. SENs are protein–polymer hybrids with a thin crosslinked mantle covering the surface of individual enzymes.^[^ ^28^, ^29^ ^]^ This polymeric mantle can be designed with either a positive or negative net charge, altering the natural surface charges of the enzymes. This modification can facilitate their strategic localization within the multilayer system through electrostatic interactions.^[^ ^30^, ^31^ ^]^ Hence, both nanozymes, that is, Mn_3_O_4_ nanoparticles, and enzyme–polymer hybrids, that is, glucose oxidase (GOx) and horseradish peroxidase (HRP)‐loaded SENs, will be explored as catalytically active building blocks as alternatives to traditional polyelectrolytes used in LbL strategy, namely poly(allylamine hydrochloride) (PAH) and poly(sodium‐p‐styrene sulfonate) (PSS) (see **Scheme** **1**). The catalytic characterization of the systems will provide new insights into the advantages of compartmentalized architecture. Finally, given the oxidant potential of the selected building blocks, the biomedical application of the GOx/Mn_3_O_4_ tandem will be assessed.

## 2 Results and Discussion

### 2.1 SENs as Building Blocks for Multilayered Membranes

#### 2.1.1 Unmodified Enzymes Show Low Particle Coverage in the LbL Approach

In this work, we aimed to exploit the potential of multilayered systems to effectively confine and position various catalytic units, thereby enhancing the synergy of multiple functionalities. Our initial focus was optimizing configurations for efficient cascade reactions based on two enzymes using LbL‐stabilized calcium carbonate, CaCO_3_, reactors. As a proof‐of‐concept, we evaluated a well‐established enzyme cascade system consisting of GOx and HRP. In this cascade, GOx catalyzes the oxidation of glucose, producing hydrogen peroxide, which HRP subsequently uses to oxidize small substrates such as 2,2′‐azinobis(3‐ethylbenzothiazoline‐6‐sulfonic acid) (ABTS).

While embedding enzymes within an inorganic core has been reported,^[^ ^32^ ^]^ we propose a more challenging approach: distributing the catalysts within the polymeric layers while maintaining the alternating charge structures typical of the LbL methodology. Due to proteins’ intricate structure and chemical composition, electrostatic immobilization is often inefficient. This ineffective interaction presents major challenges in achieving effective surface coverage, as LbL‐based systems hinder the formation of sandwich‐like arrangements that are otherwise attainable with suitable polyelectrolytes. For example, HRP and GOx showed immobilization yields of only 2.2% and 17.0%, respectively, when incubated with PAH‐coated CaCO_3_ microparticles at pH 6.5. Moreover, the ζ‐potential of modified particles (C1 and C2 in **Table** **1**) changed only slightly from +12.3 ± 2.4 to +3.4 ± 1.1 and +7.9 ± 0.5 mV upon immobilization of HRP and GOx, respectively (Table S1, Supporting Information). These results indicate that immobilizing unmodified proteins via electrostatic interactions in the LbL approach is inefficient.

**Table 1:** List of samples synthesized in this work. The composition of the template and the layered sequence are disclosed.

Nº	Template	Layer								
		#1	#2	#3	#4	#5	#6	#7	#8	#9
C1	CaCO_3_	PAH	GOx	–	–	–	–	–	–	–
C2	CaCO_3_	PAH	HRP	–	–	–	–	–	–	–
S1	CaCO_3_ + GOx	PAH	PSS	HRP@APTAC	PSS	PAH	PSS	–	–	–
S2	CaCO_3_ + GOx	PAH	PSS	PAH	PSS	HRP@APTAC	PSS	–	–	–
S3	CaCO_3_	PAH	GOx@MAEP	PAH	PSS	HRP@APTAC	PSS	–	–	–
S4	CaCO_3_	PAH	PSS	HRP@APTAC	GOx@MAEP	PAH	PSS	–	–	–
S5	CaCO_3_	PAH	PSS	HRP@APTAC	PSS	PAH	GOx@MAEP	PAH	–	–
S6a)	CaCO_3_	GOx@MAEP	HRP@APTAC	GOx@MAEP	HRP@APTAC	–	–	–	–	–
S7b)	CaCO_3_	GOx@MAEP	HRP@APTAC	GOx@MAEP	HRP@APTAC	–	–	–	–	–
S8c)	CaCO_3_	GOx@MAEP	HRP@APTAC	GOx@MAEP	HRP@APTAC	–	–	–	–	–
S9	SiO_2_ d)	PAH	GOx@MAEP	PAH	PSS	HRP@APTAC	PSS	–	–	–
S10	CaCO_3_	PSS	PAH	GOx@AAe)	Mn_3_O_4_	PSS	Mn_3_O_4_	PSS	–	–
S11	CaCO_3_	PSS	GOx@APTAC/ Mn_3_O_4_	PSS	GOx@APTAC/ Mn_3_O_4_	PSS	GOx@APTAC/ Mn_3_O_4_	PSS	GOx@APTAC/ Mn_3_O_4_	PSS

Abbreviations: APTAC, (3‐acrylamidopropyl)trimethylammonium; CaCO_3_, calcium carbonate microparticles; GOx, glucose oxidase; HRP, Horseradish peroxidase; MAEP, mono‐acryloxyethyl phosphate; Mn_3_O_4_, manganese(II,III) oxide nanoparticles; PAH, poly(allylamine hydrochloride); PSS, poly(sodium‐p‐styrene sulfonate); SiO_2_, silicon dioxide nanoparticles.

a) GOx@MAEP and HRP@APTAC loadings of 25 and 576 μg, respectively.

b) GOx@MAEP and HRP@APTAC loadings of 50 μg and 1.1 mg, respectively.

c) GOx@MAEP and HRP@APTAC loadings of 125 μg and 2.2 mg, respectively.

d) Mesoporous silica nanoparticles as template.

e) GOx SEN synthesized with AAm as anionic monomer.

In this regard, we hypothesized that engineering proteins with multivalent charged groups on their surface could strengthen electrostatic interactions and thereby increase the protein loading within multilayered systems. Enzyme‐polymer hybrids represent ideal catalytic building blocks in this approach. Specifically, the SEN strategy involves fully coated enzymes with charged polymers, providing sufficient surface charge to facilitate effective electrostatic assembly and positioning them as functional, catalytically active alternatives to conventional polyelectrolytes. Therefore, we explored using SENs, GOx@SEN, and HRP@SEN as alternatives to PAH and PSS polyelectrolytes. Negatively charged GOx nanogels (GOx@MAEP) were synthesized using monoacryloxyethyl phosphate, and positively charged HRP nanogels were prepared using (3‐acrylamidopropyl) trimethylammonium chloride (HRP@APTAC). These nanogels were synthesized to replace PAH and PSS as building blocks (details on the synthesis and characterization are provided in Table S2 and S3 and Figure S1–S5, Supporting Information). Notably, this strategy resulted in a full shift in the ζ‐potential of HRP enzyme (isoelectric point of 4.2–4.5) from −11.4 ± 1.2 mV in the native enzyme to +7.4 ± 0.8 mV in the hybrid form (Table S1, Supporting Information).

#### 2.1.2 Bi‐Enzymatic Microparticles: Confinement in the Core and the Membrane

**Figure 1:**
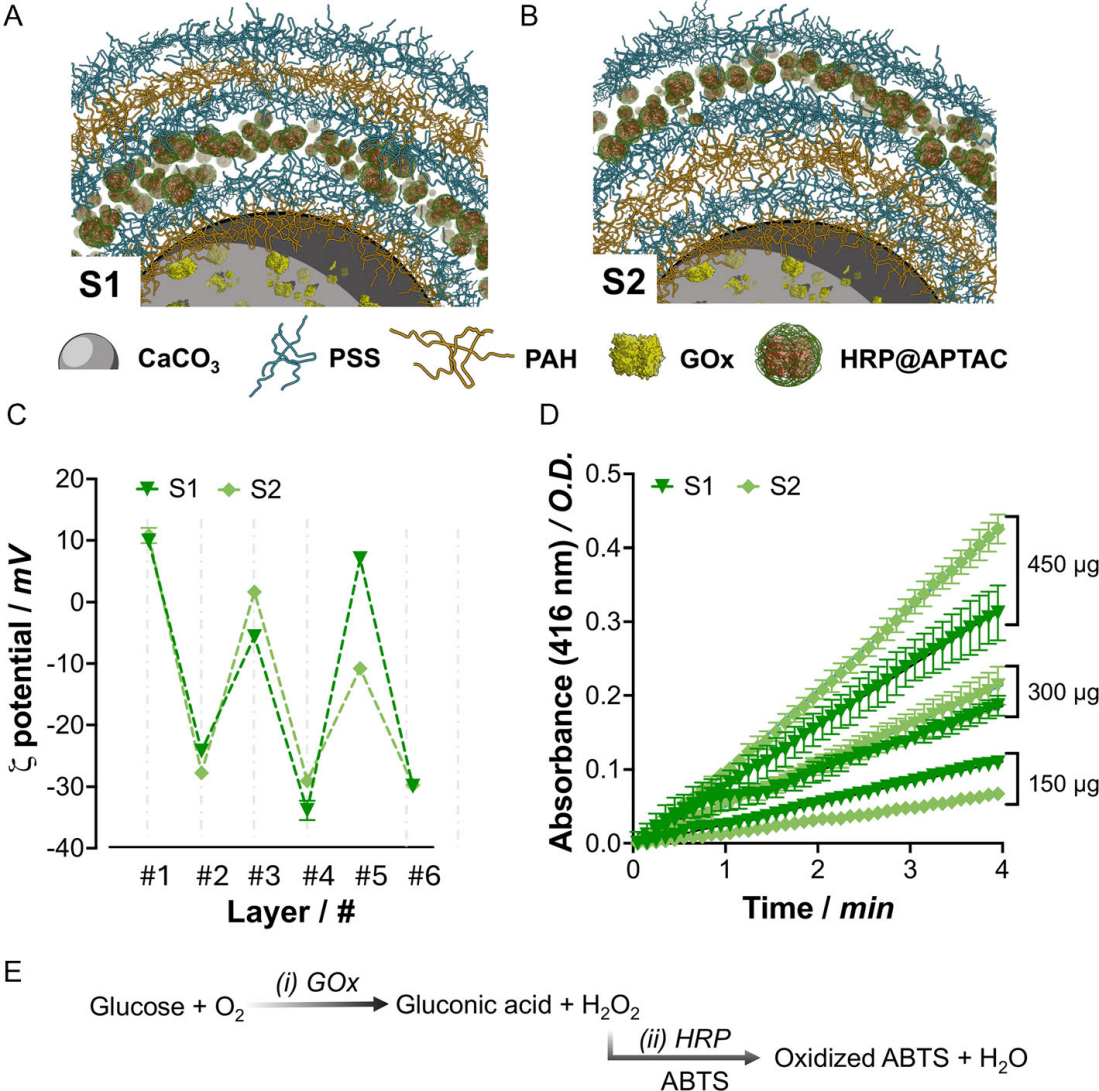
Design and characterization of S1 and S2. A,B) Schematic depiction of enzyme‐loaded CaCO_3_ microparticles with a multilayered membrane, where HRP@APTAC hybrids are incorporated either in layer #3 (S1, A) or layer #5 (S2, B). C) ζ‐potential values measured after each layer deposition for S1 and S2. D) Kinetic measurements were performed for S1 and S2 using increasing particle amounts (150, 300, and 450 μg). E) Schematic representation of the catalytic reaction mechanism involved in the enzymatic cascade reaction monitored in this study. Data are presented as mean±SD from technical triplicates (*n* = 3).

The design of compartmentalized bi‐enzymatic reactors within CaCO_3_ microparticles enables various configurations. In our initial approach, we conducted experiments with free GOx encapsulated in the core to assess the effect of replacing PAH with HRP@APTAC in layers #3 or #5. The optimal sequence was determined based on the multilayer formation efficiency, assessed by ζ‐potential shifts, and its impact on cascade reaction performance was evaluated through kinetic catalytic measurements. Thus, we fabricated samples S1 and S2, incorporating HRP@APTAC into layers #3 and #5, respectively (see Table 1 and the schematic representation of the arranged sequence in **Figure** **1**A,B).

Incorporating GOx into the core was relatively efficient, with 93.5% of the seeded enzyme successfully entrapped. GOx/CaCO_3_ particles, with a ζ‐potential of *ca*. −1.5 mV, show a significant increase to *ca*. +10.0 mV upon the deposition of PAH. Interestingly, compared to the results obtained with free HRP, HRP@APTAC achieved an immobilization yield of nearly 100%, regardless of the layer in which it was deposited. When comparing S1 and S2, similar ζ‐potential values were measured for layer #3, where HRP@APTAC and PAH were deposited, respectively (Figure 1C). Subsequent deposition of PSS resulted in a similar ζ‐potential decrease (−29.2 for S1 and −21.9 mV for S2). These results demonstrate that HRP@APTAC can be used as a building block for synthesizing multilayered materials. However, the surface coverage of the HRP@APTAC building block in layer #5 of S2 appears less efficient, as indicated by the smaller ζ‐potential shift in Figure 1C (a shift of +40 mV for S1 vs. +19 mV for S2). This reduced efficiency may result from the larger area covered in layer #5 compared to layer #3. Nevertheless, the typical zigzag behavior of the ζ‐potential profiles is still observed in both samples, suggesting the successful incorporation of HRP@APTAC as layer #3 or #5 in S1 and S2, respectively.

Comparing the catalytic performance of S1 and S2 allows us to investigate the role of enzyme allocation in a compartmentalized system. On the one hand, S1 positions both catalytic entities close together (only two layers apart), which may enhance cascade efficiency by minimizing the distance to be crossed by the reaction intermediates. Conversely, S2, despite having a greater distance between the enzymes, features HRP in a more accessible layer, potentially improving substrate access, specifically ABTS. Catalytic measurements were conducted by monitoring ABTS oxidation at 416 nm (Figure 1D,E). Glucose consumption was calculated assuming an equimolar ratio of processed glucose to oxidized ABTS (see Supporting Information for details). The diffusion effect was evident when evaluating the activity of S1 and S2 at various particle concentrations. At high amounts (450 μg), the activity of S2 was significantly greater than that of S1 (1.232 ± 0.005 vs. 0.889 ± 0.003 nmol of glucose min^−1^, respectively), suggesting that placing the HRP enzyme in the microparticles’ outer layers may provide the most effective configuration. When the particle amount was decreased to 300 μg, the difference between the kinetic profiles was reduced to 0.498 ± 0.007 vs. 0.580 ± 0.002 nmol of glucose min^−1^ for S1 and S2, respectively. Notably, when added particles were further reduced (150 μg), S1 outperformed S2 by 164% with 0.316 ± 0.002 vs. 0.193 ± 0.002 nmol of glucose min^−1^, respectively.

These results indicate that close enzyme localization at lower hydrogen peroxide levels is critical to achieving effective cascade reactions mimicking the metabolic channeling observed in living cells. Hence, the loss of intermediates is minimized, and the efficiency of the overall reaction is maximized. This behavior has been previously observed in other multicatalytic systems where functional units were confined to separate compartments.^[^ ^13^ ^]^

#### 2.1.3 Bi‐Enzymatic Microcapsules: Confinement in the Core and the Membrane

The entrapment of enzymes inside the inorganic templates is inherently linked with the reduction of enzymatic performance due to diffusion issues. For this reason, researchers have systematically removed the core of polymer‐stabilized inorganic particles,^[^ ^32^ ^]^ forming hollow microcapsules with a multilayered membrane.^[^ ^33^ ^]^ In the case of CaCO_3_ particles, cation complexation agents such as EDTA are used.^[^ ^34^ ^]^ Typically, concentrations that range between 20 and 100 mM are used. However, when these conditions were applied to our system, we observed that, while the structural integrity of the particles was mainly conserved, the cascade reaction was depleted (see Figure S6, Supporting Information, for details). This activity loss was attributed to EDTA's detrimental effect on the catalytic performance of the GOx enzyme, in which activity dropped by 95% and 55% after incubation with 100 and 60 mM of EDTA, respectively. Alternative protocols involving overnight dialysis with a lower concentration of EDTA (i.e., 20 mM) have been explored to preserve enzyme activity. However, these prolonged incubation times combined with inadequate stirring usually lead to microcapsule aggregation and a further decline in enzyme performance.^[^ ^35^ ^]^

### 2.2 Bi‐Enzymatic Systems within Multilayered Membranes

#### 2.2.1 GOx and HRP‐Hybrids in Alternate Layers

**Figure 2:**
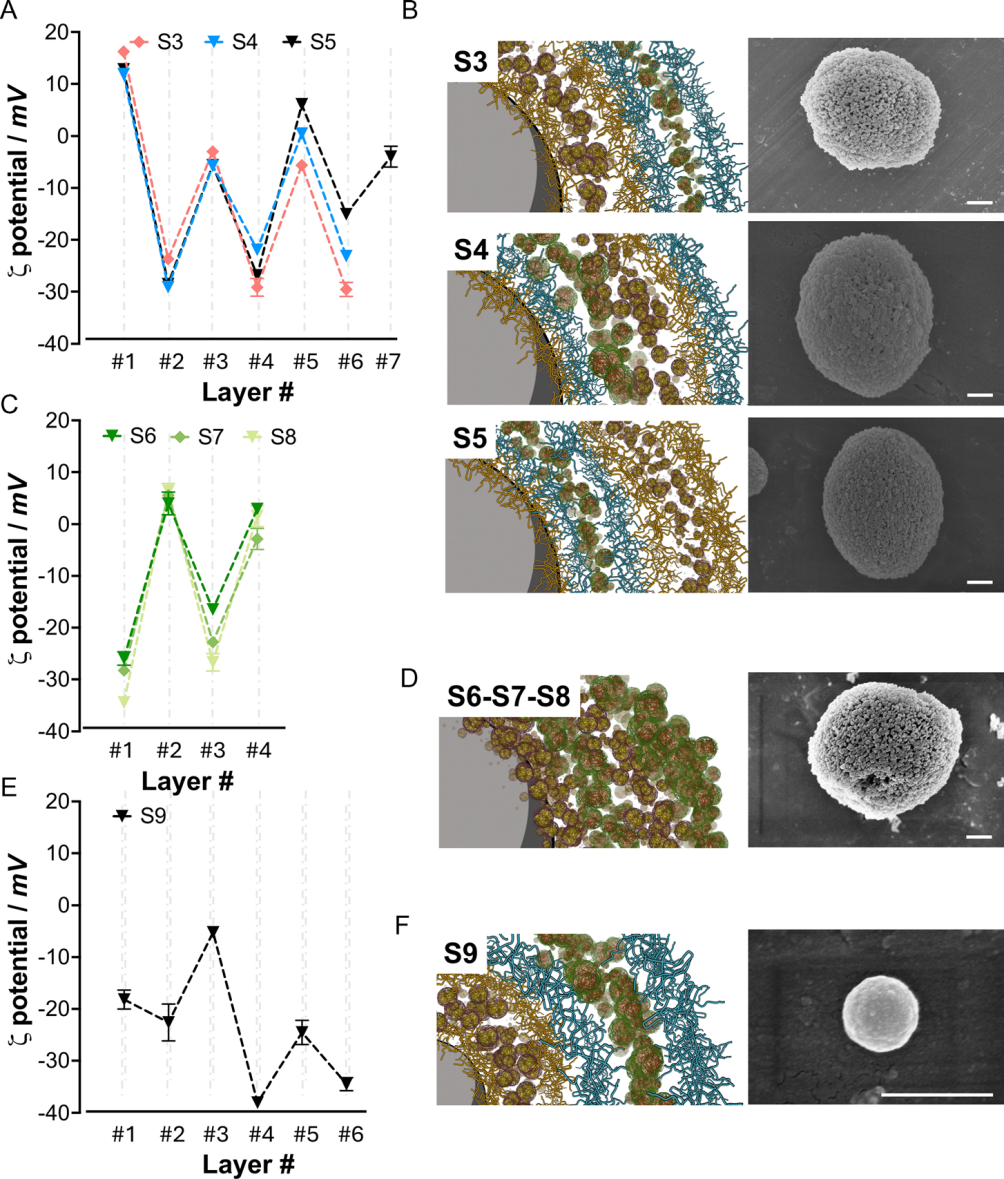
ζ‐Potential measurements (left column: A,C, and E) illustrating the surface charge changes during the LbL assembly of membranes for samples S3–S9. Corresponding schematic depictions of the multilayered membrane architecture and SEM images (right column: B,D, and F) are shown for each sample. Scale bars: 0.5 μm. Data are presented as mean±SD from technical triplicates (*n* = 3).

The results prompted the exploration of new strategies for localizing both enzymes within the material to support the diffusion of substrates, intermediates, and (by)products. Our initial findings indicate that protein–polymer hybrids could serve as viable substitutes for conventional polyelectrolytes in constructing multilayered structures, which may be useful in mimicking cell membranes of polymer‐based artificial cells.^[^ ^36^ ^]^ Hence, we fabricated an alternative sample, S3, in which the bi‐enzymatic reactor is solely deposited in the membrane, confined in distinct layers (compartments). S3 was therefore synthesized using the same LbL methodology, with GOx@MAEP introduced at layer #2 and HRP@APTAC at layer #5 (sequence disclosed in Table 1). CaCO_3_ particles, with a ζ‐potential range between −10 and −13 mV, featured a significant increase of the ζ‐potential to values between +10.9 and +12.0 mV upon the deposition of PAH. Notably, the incorporation of the two protein hybrids into the membrane, that is, layers #2 and #5, yielded similar ζ‐potential values compared to those obtained with the corresponding polyelectrolytes, PAH in layer #3 and PSS in layers #4 and #6 (**Figure** **2**A). Other configurations of bi‐enzymatic layered membranes were developed, namely S4 and S5. S4 features sequential HRP@APTAC/GOx@MAEP layers that would minimize the distance between the two enzymes, likely minimizing the diffusion of the intermediates within the membrane (schematic representations are shown in Figure 2B). Finally, S5 was designed to evaluate the deposited sequence's effect on the particles’ catalytic performance. Unlike S3, HRP@APTAC was placed in the inner layers of the membrane in S5, while GOx@MAEP was confined in the outer layer. A layer #7 was included to prevent direct exposure of the GOx@MAEP building block to the external environment. ζ‐potential values measured for S3, S4, and S5 during each assembly step are shown in Figure 2A. As observed, no significant differences were detected when building blocks were used instead of the polyelectrolytes. Moreover, SEM images of the modified particles showed no significant changes in morphology compared to CaCO_3_ templates (Figure 2B).

#### 2.2.2 Multilayered Membranes in the Absence of Noncatalytic Polyelectrolytes

With the successful fabrication of S3, S4, and S5, an à‐la‐carte design and efficient fabrication of multicatalytic layered membranes have been demonstrated. While this approach enables the sequenced allocation of compartments with assorted enzymes, several layers of polyelectrolytes were deposited to ensure the robustness and stability of the assembled membrane. However, this approach limits the loading of functional and catalytic units within the membrane. To maximize the enzyme loadings, we explored the possibility of removing the deposition of the polyelectrolytes, that is, PAH and PSS, to the membrane, utilizing only the enzyme hybrids as charged materials to construct the multilayered membranes. Hence, we synthesized S6 (Table 1). In the first attempt, HRP@APTAC was used as a substitute for PAH in layer #1.

Nevertheless, the ζ‐potential values indicated a poor deposition of the hybrids on the surface of CaCO_3_ (data not shown). For this reason, we decided to start the assembly of the membrane from the negatively charged building block, that is, GOx@MAEP. A clear shift of the ζ‐potential could now be observed (Figure 2C). Thereafter, layers #2, #4, and #3 were assembled using HRP@APTAC and GOx@MAEP as building blocks, respectively. The ζ‐potential profile confirmed the successful assembly. Using the same approach, S7 and S8 samples were synthesized by increasing the enzyme‐hybrid amount added to each layer from 600 μg to 2.3 mg. Interestingly, the ζ‐potential profile observed in Figure 2C, obtained without polyelectrolytes, resembles that obtained for S3–S5 (Figure 2A), in which PAH and PSS were introduced to enhance the membrane robustness and cohesion. Further, SEM images (Figure 2D and Figure S7, Supporting Information) show particles of ≈3 μm that exhibit the typical morphology of CaCO_3_ vaterite microparticles.

#### 2.2.3 Catalytic Performance of the Multilayered Membranes

Responsive artificial cells with functional/catalytic membranes require an efficient placement of enzymes that work together in cascade reactions. Hence, the intraparticle diffusion supporting the formation of metabolic channels or the accessibility to the external environment are key parameters that necessitate optimization. The proposed on‐demand localization of enzymes could address those parameters. Therefore, the catalytic performance of the synthesized particles was evaluated to determine the optimal configurations. In this case, the efficiency of the cascade reaction when localizing either HRP or GOx in external layers is tested. Hence, S3 and S5 displayed HRP@APTAC and GOx@MAEP in layers #5 and #6, respectively. Interestingly, S3 outperformed the activity shown by S5 by 2.3 times, suggesting the convenience of exposing the HRP enzyme close to the outermost layer of the membrane.

**Figure 3:**
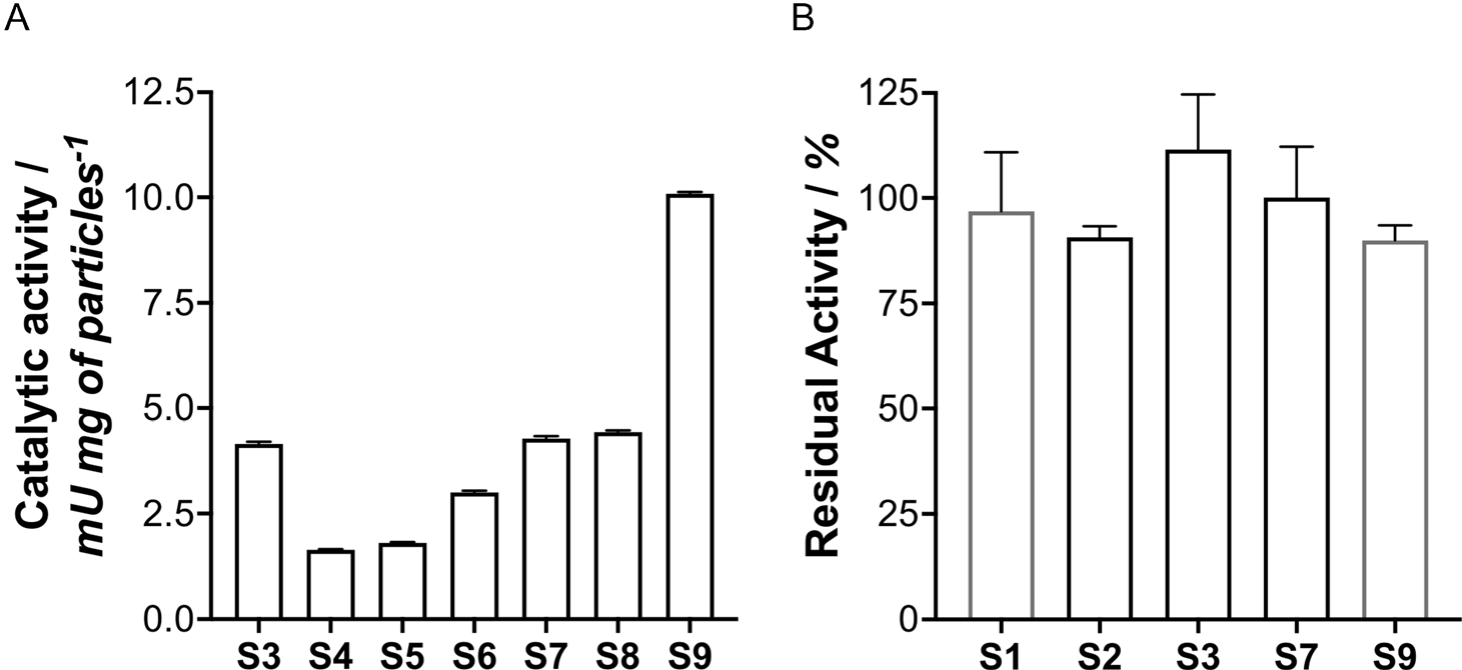
Kinetic measurements of the fabricated particles. A) Kinetic measurements reported as mU per mg of particles. B) Residual activity was measured for the microparticles after 5 days of incubation at 37 °C. 100% of the activity is referred to as the activity of freshly prepared particles. Data are presented as mean±SD from technical triplicates (*n* = 3).

After measuring catalytic activity, we observed no significant differences between samples S4 and S5. However, both exhibited a reduced activity of ≈60% compared to sample S3 (**Figure** **3**A). These findings suggest that the exposure of GOx to substrates in external layers is not conducive to efficient cascade reactions. In contrast, positioning GOx in more internal layers yields an optimal arrangement. This phenomenon can be explained by examining the diffusion behavior of the reaction intermediates, particularly in hydrogen peroxide (H_2_O_2_). Given that H_2_O_2_ is a small molecule, its outward diffusion from the microparticle may compromise the efficiency of peroxidation. Conversely, the internalization of GOx implies that the H_2_O_2_, during its diffusion toward the external environment, is likely utilized by HRP to oxidize ABTS. This same diffusion issue also affects ABTS, a relatively large molecule that must traverse several layers (3–4) to reach the HRP in samples S4 and S5. Hence, these diffusion issues may explain the observed reduction in activity in samples S4 and S5.

Samples S6 to S8 were synthesized without PSS and PAH polyelectrolytes. Interestingly, to reach the highest activity with this architecture, the enzyme hybrid loads had to be increased from 25 to 50 μg of GOx@MAEP and from 576 μg to 1.1 mg of HRP@APTAC. When higher amounts were seeded, that is, 125 μg of GOx@MAEP and 2.2 mg of HRP@APTAC, the same activity was achieved (Figure 3A), suggesting that the surface saturation was reached in sample S7. Interestingly, the measured activity was like that exhibited by the best configuration, that is, S3, in previous systems.

#### 2.2.4 Versatile Approach: Use of Silica Nanoparticles

We showed that enzyme hybrids can build catalytic membranes with well‐organized layers. Next, we explored whether a similar approach could fabricate membranes on the surface of nanosized particles. Due to the strong potential in biomedical applications,^[^ ^37^ ^]^ we tested the convenience of mesoporous silica nanoparticles as inorganic colloidal substrates of ≈200 nm to be covered by enzyme‐hybrid membranes. We tested the best configuration achieved for CaCO_3_ microparticles, namely the sequence used for S3, to assess the versatility and limitations of our approach in modifying nanomaterials. The synthesized material, referred to as S9, was prepared using the same enzyme hybrid concentrations as those used for S3, and it showed the typical zigzag‐shaped ζ‐potential profile measured upon each of the layer deposition steps (Figure 2E and schematic depiction of the sequence and scanning electron microscopy (SEM) image shown in Figure 2F). The potential shifts were less pronounced than those observed for S3, likely due to the reduced surface area. Interestingly, the reduction in the size of the modified nanomaterial and, consequently, the scale‐down of the membrane dimensions led to a twofold enhancement in the catalytic performance of the cascade reaction compared to S3 (Figure 3A). These results are highly relevant as they suggest that, besides the layer‐to‐layer distance, the scale at which the enzymes are compartmentalized is critical when designing multicatalytic systems. Implementing smaller confinements may increase the effective substrate concentrations near immobilized enzymes,^[^ ^38^ ^]^ a phenomenon often accompanied by enhanced catalytic performance.^[^ ^39^ ^]^

#### 2.2.5 Stability of the Formulated Systems

The shelf‐stability of the developed formulations was analyzed by assessing their catalytic activity and morphology upon storage. Given the particles’ potential for biomedical applications, a temperature of 37 °C and 5 days of incubation were chosen to assess their stability. These conditions are comparable to those found in routine in vitro cell experiments. It is important to note that naked vaterite CaCO_3_ microparticles usually undergo an allotropic transformation to the more stable calcite due to their thermodynamic instability,^[^ ^40^ ^]^ which can be prevented by coating them with polymeric layers. Therefore, the conservation of CaCO_3_ vaterite structure would indicate robust assembled membranes.

In all the systems, the typical spherical and spongy structure of vaterite was preserved for up to 5 days, and no signs of transformation toward calcite were observed (Figure S8, Supporting Information). Moreover, catalytic measurements indicate the conservation of the functionality after the incubation (Figure 3B). Thus, we conclude that coating vaterite CaCO_3_ microparticles with enzyme–polymer hybrids is sufficient for stabilizing their crystalline structure, even at the lowest tested concentration. The stability of the S9 system prepared using mesoporous silica nanoparticles as a core was also assessed by SEM and catalytic‐wise. In this case, no significant morphological changes were observed after incubation, suggesting good structural stability of the formulation.

### 2.3 Design of Bifunctional Organic–Inorganic Microreactors

#### 2.3.1 Assembly of Mn_3_O_4_ Nanoparticles on the Multilayered CaCO_3_ Template

As discussed in the previous sections, the LbL approach allowed us to fabricate highly versatile colloidal reactors with precise configurations at the nano‐ and microscale. The considered building blocks, SENs, could be assembled ad hoc, thanks to the engineered enzymes’ robustness and tunable surface charge, which facilitates electrostatic interactions with other polyelectrolytes. The explored approach also allowed the minimization of non‐reactive entities (e.g., CaCO_3_ template, and polyelectrolytes), resulting in efficient nano‐ and microreactors for enzymatic cascade reactions.

To further challenge the versatility of the LbL approach, we explored the possibility of broadening the membrane composition by introducing inorganic nanoparticles, giving rise to layered bifunctional organic–inorganic membranes. This strategy introduces new possibilities in artificial membrane design with built‐in functionalities, including incorporating catalytic nanozymes. For example, incorporating Mn_3_O_4_ nanozymes could result in assembled membranes with therapeutic activity by interfering with the redox balance of cancer cells.^[^ ^21^, ^41^ ^]^ Other examples in the literature include the embedding of nanozymes, such as ultrasmall gold nanoparticles or hemin moieties, into porous resins and MOFs, as well as the in situ growth of catalytic nanozymes (e.g., platinum nanoparticles) on the surface of microparticles to develop efficient biosensing systems.^[^ ^42^, ^43^ ^]^

Here, we used Mn_3_O_4_ nanozymes^[^ ^44^ ^]^ as a proof‐of‐concept to explore the incorporation of inorganic nanoparticles within the LbL process alongside natural enzymes, such as GOx; this approach enabled the assembly of organic‐inorganic artificial membranes, where GOx/Mn_3_O_4_ catalytic unitswork synergistically. In the presence of glucose*,* GOx generates hydrogen peroxide in situ, which is then utilized by Mn_3_O_4_ nanozymes to disrupt the oxidative balance in cellular metabolism through the catalytic reaction of the produced H_2_O_2_.^[^ ^20^, ^45^ ^]^ Mn_3_O_4_ nanoparticles were synthesized following a multistep process that comprises the reaction between KMnO_4_ and PAH (details on the synthesis and characterization of the Mn_3_O_4_ are shown in Supporting Information in Figure S9–S11 and Table S4). This process yields a highly crystalline nanomaterial with a spherical shape (Figure S12, Supporting Information), hydrodynamic diameter of 61.10 ± 14.05 nm, and a surface ζ‐potential of +31.6 ± 7.8 mV (Figure S13, Supporting Information), which can be ascribed to the presence of PAH during the synthesis protocol. Thereafter, two approaches were followed to assemble these Mn_3_O_4_ nanoparticles on CaCO_3_ microparticles following the LbL approach: i) membranes with confined enzymes and nanozymes in alternate layers and ii) membranes with hybrid enzyme/nanozyme layers. Both methods are described in the following sections.

##### Membranes with Confined Enzymes and Nanozymes in Alternate Layers

**Figure 4:**
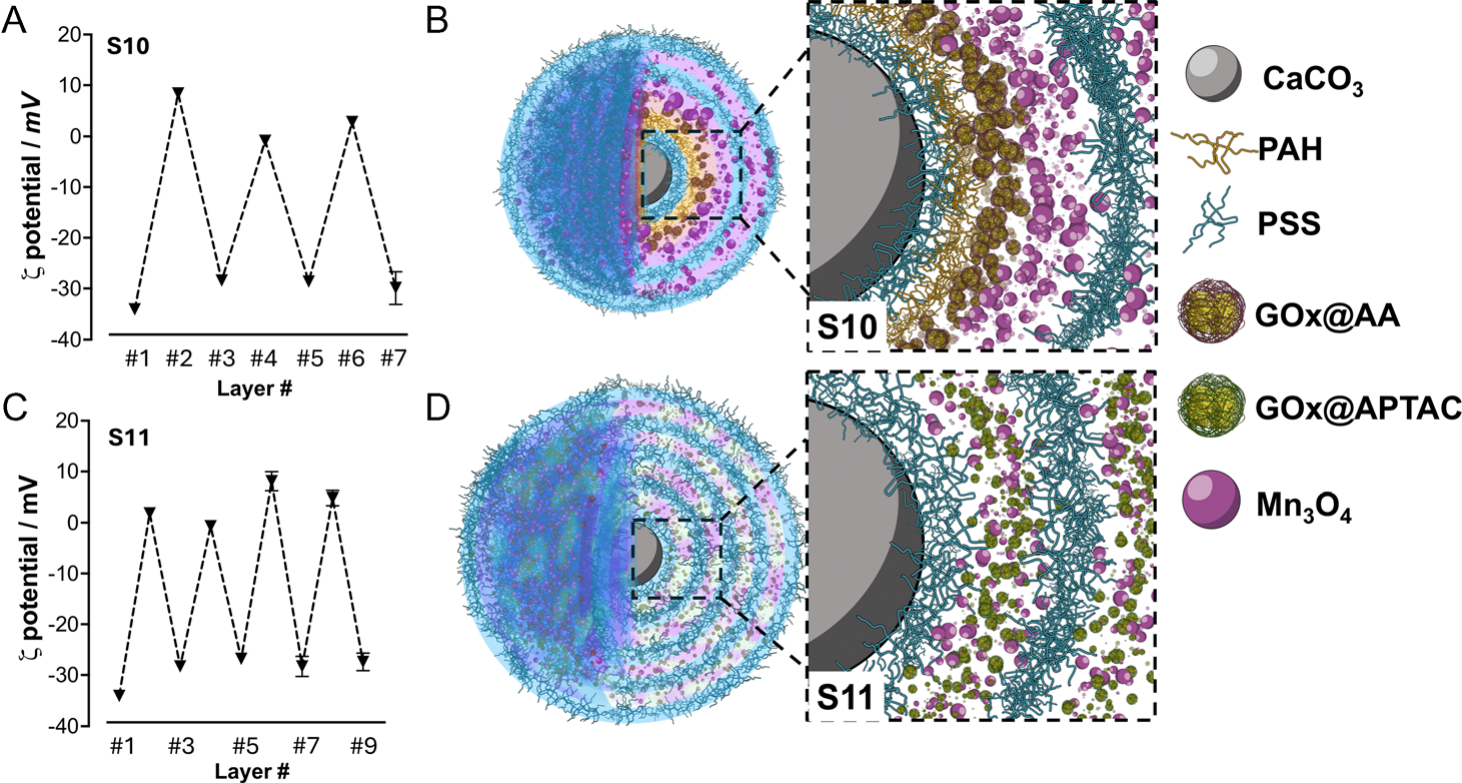
Measured ζ‐potential profile and schematic depiction of the multilayered membrane of A,B) S10 and C,D) S11. Data are presented as mean±SD from technical triplicates (*n* = 3).

In this set of experiments, we further tested the possibility of exchanging the composition of the polymeric nanogel that wraps the GOx in SENs. Instead of using monoacryloxyethyl phosphate (MAEP) monomer to achieve negatively charged SENs, we synthesized a new batch of GOx‐loaded SENs using acrylic acid as an ionic monomer (synthesis and characterization details are shown in the Supporting Information in Table S1–S3 and Figure S1, S2, S4, and S5). These enzyme hybrids were designated as GOx@AA and showed promising features (hydrodynamic diameter of 9.2 ± 1.1 nm and ζ‐potential of −33.26 ± 1.59 mV) to be used as building blocks in LbL‐based artificial membranes. Interestingly, the potential use of distinct polymeric compositions was demonstrated with the successful deposition—immobilization yield of 100%—of GOx@AA in layer #3 as a negatively charged building block (**Figure** **4**A). This configuration was selected based on our previous results (see Section 2.2.1), suggesting that GOx in internal layers represents an optimal arrangement for enzymatic cascade reactions. Mn_3_O_4_ nanoparticles were incorporated in layer #4 as a positively charged building block. However, when Mn_3_O_4_ nanoparticles were incubated with the 3‐layer CaCO_3_ particles, instantaneous flocculation of the Mn_3_O_4_ nanoparticles was observed, and their assembly was precluded. This issue could be overcome by the co‐addition of PAH with Mn_3_O_4_ (see Supporting Information).

We hypothesize that the sole addition of Mn_3_O_4_ was not sufficient for the complete coverage of the membrane, and therefore, the flocculation of the system was triggered. The co‐addition of PAH would stabilize the Mn_3_O_4_ particles and block the negatively charged patches uncovered by Mn_3_O_4_. This strategy enables the introduction of ≈40 μg of Mn_3_O_4_ in the layer #4 (Figure S14, Supporting Information). As our previous measurements indicated that higher amounts of nanozyme were needed for significant catalysis, we repeated the deposition of Mn_3_O_4_ in layer #6. Interestingly, the Mn_3_O_4_ load could be increased to ≈120 μg, probably due to the significantly increased area of the membrane. Therefore, we achieved total loadings of 162.1 μg of Mn_3_O_4_ nanoparticles and 8.98 μg of GOx per mg of CaCO_3_ microparticles. Both ζ‐potential values and Fourier‐transform infrared (FTIR) spectra (Figure S15, Supporting Information) suggested the correct assembly of the building blocks in a sample assigned as S10 (Table 1 and Figure 4B).

##### Co‐Immobilization of Enzyme and Nanozyme Entities

In a second approach, we designed hybrid membranes in which SENs and nanozymes are confined in the same layers, giving rise to S11. This configuration was selected to maximize the number of catalytic entities and ensure their proximity, thereby facilitating the potential enzymatic cascade reaction. To accomplish this, GOx was wrapped within a positively charged nanogel. We synthesized GOx@APTAC enzyme‐polymer hybrid by utilizing (3‐acrylamidopropyl) trimethylammonium chloride as a cationic polymer (see Table S1–S3 and Figure S1, S2, S4, and S5, Supporting Information). As in the case of HRP@APTAC, GOx@APTAC hybrids display a complete shift of the surface potential—from −14.6 ± 0.5 to +27.6 ± 0.9 mV measured for unmodified GOx and GOx@APTAC, respectively (Table S1, Supporting Information).

Herein, we achieved hybrid layers in #2, #4, #6, and #8. As explained in the Supporting Information, this was achieved by co‐incubating the Mn_3_O_4_ nanoparticles and GOx@APTAC with the template in the presence of PAH to prevent nanoparticle flocculation. The assembly of the building blocks occurred satisfactorily, as evidenced by the clear shift in the ζ‐potential values after each deposition step (Figure 4C) and new bands corresponding to the building blocks appearing in the FT‐IR spectra (Figure S15, Supporting Information). Several layers were deposited due to the reduced deposition efficiency observed with this strategy. We hypothesize that the co‐incubation of three positively charged building blocks (i.e., PAH, Mn_3_O_4_ nanoparticles, and GOx@APTAC) may have led to an electrostatic repulsion, negatively impacting incorporation efficiency. Hence, after four deposition steps, a loading of 121.5 and 9.39 μg of Mn_3_O_4_ and GOx@APTAC per mg of CaCO_3_ microparticles was achieved, respectively.

#### 2.3.2 Characterization of the Activity on Organic–Inorganic Microreactors

**Figure 5:**
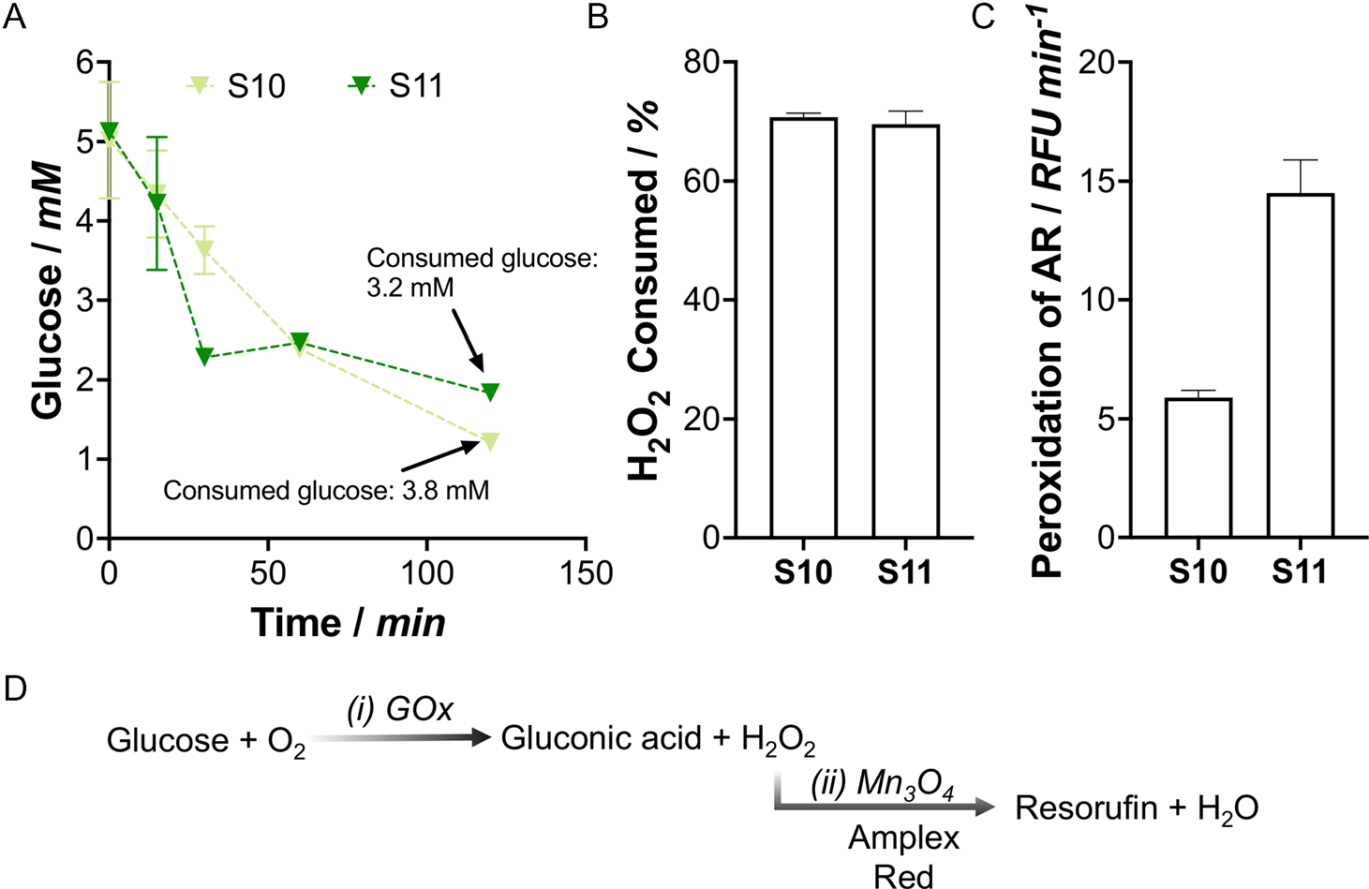
Catalytic characterization of organic‐inorganic microreactors. A) Glucose consumption was measured for S10 and S11 by the 3,5‐dinitrosalicylic acid (DNS) assay. B) Catalase‐like activity measured for S10 and S11. C) Cascade reaction performance assessed by Amplex Red (AR) peroxidation in the presence of glucose for S10 and S11. D) Schematic representation of the catalytic mechanism underlying the cascade reaction shown in graph (C). Data are presented as mean±SD from technical triplicates (*n* = 3).

Given the complexity of the organic‐inorganic systems developed here, comprising natural and artificial enzymes with glucose oxidase (GOx) and catalase‐like and peroxidase‐like (Mn_3_O_4_) activity, we first evaluated their individual catalytic performances. We initially tested the glucose consumption ability of S10 and S11 by incubating samples in a 5 mM aqueous glucose solution and quantifying the remaining concentration at different time points (Figure S16, Supporting Information). As observed in **Figure** **5**A, both formulations of microreactors efficiently reduced the glucose concentration from the solution with similar performance, indicating that GOx@AA and GOx@APTAC were active within the multilayer membrane.

We next investigated the microreactors’ catalase‐like activity (Figure 5B), which can be attributed to the presence of Mn_3_O_4_ nanoparticles, as previously reported.^[^ ^41^ ^]^ Different microreactor formulations were incubated with 50 μM H_2_O_2_; the remaining concentration was quantified after 15 min. The microreactors showed significant catalase‐like activity, with >75% of the H_2_O_2_ removed after 15 min. The catalase‐like activity of the nanozymes was confirmed after the assembly process, yet no significant differences were observed between S10 and S11.

Finally, we wanted to prove the peroxidation capability of the microreactors. The combined integration of GOx and Mn_3_O_4_ is presented as a one‐pot concurrent cascade reaction composed of hybrid (organic–inorganic) components confined in tailored membranes. In the presence of glucose, GOx produces hydrogen peroxide in situ, which is further used by Mn_3_O_4_ nanozymes to peroxidize small compounds.^[^ ^46^ ^]^ To test this activity, the microreactors were incubated with glucose in the presence of Amplex Red (AR), whose oxidation can be monitored by the increase of fluorescence at 585 nm due to the formation of resorufin (details in Supporting Information) (Figure 5C,D). While control samples showed no signal, S10 and S11 completed the cascade reaction. We found that the cascade reaction of S11 was 2.5‐fold more efficient despite having a lower Mn_3_O_4_ load (121.5 vs. 162.1 μg of Mn_3_O_4_ for S11 and S10, respectively). This result highlights the importance of catalytic co‐localization in maximizing performance. Moreover, this experiment demonstrates the feasibility of integrating organic and inorganic entities within the same colloidal template to perform enzymatic cascade reactions.

### 2.4 In Vitro Assessment

**Figure 6:**
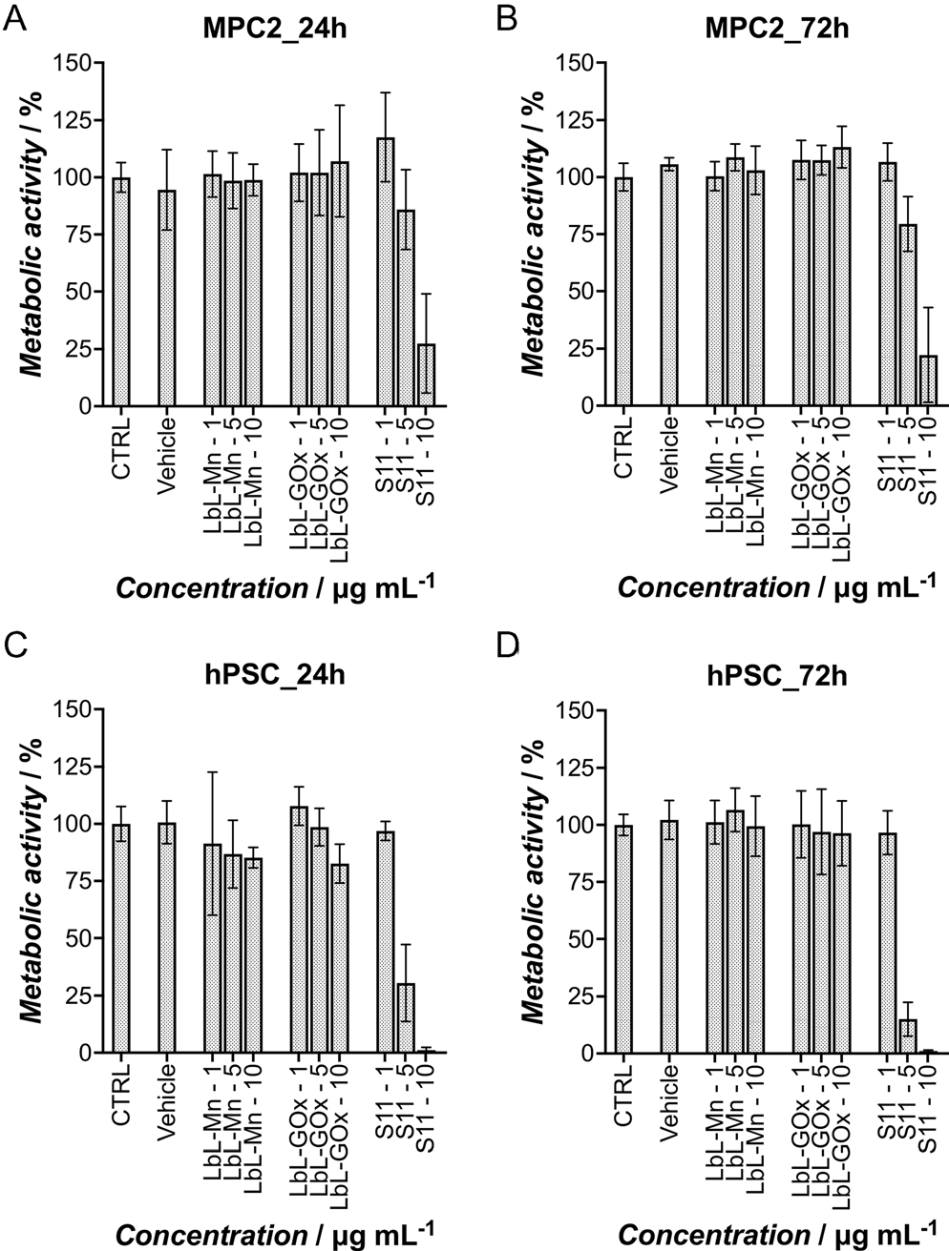
Normalized metabolic activity of human MiaPaCa‐2 (MPC2) cells and human pancreatic stellate cells (hPSCs) at 24 and 72 h. Panels A) and B) display the relative metabolic activity of MPC2 cells at 24 and 72 h, respectively, while panels C) and D) show the corresponding data for hPSCs. The *x*‐axis labels represent different treatments: vehicle: the highest amount (10% v/v) of phosphate buffer saline used to perform the required dilutions, LbL‐Mn (layer‐by‐layer microcapsules containing only Mn_3_O_4_ nanoparticles), LbL‐GOx (microcapsules containing only glucose oxidase), and S11 (microcapsules containing both Mn_3_O_4_ and glucose oxidase). Numbers 1, 5, and 10 next to the names represent the concentrations 1, 5, and 10 μg mL^−1^ for each sample. All samples were normalized to the average value of the control group. Statistical significance is detailed in Tables S5a, S5b, S6a, and S6b (see Supporting Information), with *p* values provided for each comparison. Data are presented as mean±SD from three individual experiments (*N* = 3).

In this preliminary biological study (**Figure** **6**), we evaluated the effects of LbL microcapsules containing Mn_3_O_4_ nanoparticles (LbL‐Mn), GOx alone (LbL‐GOx), or a combination of both (S11, Table 1) on the metabolic activity of 2D cell monolayers of human MiaPaCa‐2 (MPC2) cancer cells (Figure 6A,B) and human pancreatic stellate cells (hPSCs) (Figure 6C,D). LbL‐Mn and LbL‐GOx were used as controls for S11, representing systems with single catalytic activity in contrast to the multicatalytic activity of S11.

The results showed that the combinatorial approach, S11, significantly reduced cellular metabolism compared to treatments with the individual components (LbL‐Mn or LbL‐GOx), with more pronounced effects observed in hPSCs. Specifically, in hPSCs (Table S5b, Supporting Information), significant reductions were recorded at 24 h (***p < 0.001 at 5 μg mL^−1^ and ****p < 0.0001 at 10 μg mL^−1^) and 72 h (****p < 0.0001 at both 5 and 10 μg mL^−1^). In MPC2 cells, no significant effect (“ns”) was noted at 5 μg mL^−1^ at 24 h. Nevertheless, a moderate reduction occurred at 10 μg mL^−1^ (**p < 0.01), while at 72 h, metabolic activity was slightly reduced at 5 μg mL^−1^ (*p < 0.05) and more significantly at 10 μg mL^−1^ (***p < 0.001). In contrast, treatments with either Mn_3_O_4_ (LbL‐Mn) or GOx alone (LbL‐GOx) showed no significant effects across all conditions tested.

These findings highlight the superior efficacy of the combinatorial approach, particularly in hPSCs. While Mn_3_O_4_ nanozymes exhibit oxidase‐like activity,^[^ ^45^, ^47^ ^]^ leading to the production of reactive oxygen species (ROS), GOx catalyzes the glucose oxidation to gluconic acid and hydrogen peroxide, further contributing to ROS generation. Elevated ROS levels can induce oxidative stress in both cell types, potentially leading to reduced metabolic activity and cell death. Additionally, Mn_3_O_4_ could facilitate oxidative modifications in other biomolecules, further amplifying cellular stress responses. Given that Mn_3_O_4_ utilizes GOx‐generated H_2_O_2_ as a reactive substrate in S11, this system may trigger lipid peroxidation, compromising membrane integrity and cell function. However, Mn_3_O_4_ also exhibits catalase‐like activity, which can scavenge H_2_O_2_ and mitigate oxidative stress. Thus, the observed reduction in cellular metabolism likely arises from a balance between ROS generation and scavenging, with peroxidation activity playing a key role in disrupting oxidative homeostasis.

The differences between hPSCs and MPC2 may be attributed to variations in their susceptibility to oxidative stress and differences in their metabolic pathways. hPSCs play a critical role in pancreatic cancer by promoting tumor growth, invasion, and resistance to therapy through their activation, that is, the transition of hPSCs from a quiescent to a myofibroblast‐like state, characterized by increased production of extracellular matrix proteins and secretion of growth factors and cytokines.^[^ ^48^ ^]^ Therefore, their enhanced sensitivity to S11 (LbL‐GOx‐Mn) treatment, as presented in Figure 6A–D, could modulate their activation state, potentially disrupting the tumor stroma and influencing cancer progression. In contrast, cancer cells like MPC2 often exhibit enhanced antioxidant defenses, allowing them to tolerate increased ROS levels.^[^ ^49^ ^]^ This adaptability may explain the reduced impact of S11 on MPC2 cells, underscoring the potential need for combination therapies to target cancer cells and the supportive tumor microenvironment effectively.

Finally, we acknowledge the limitation that 2D cell monolayers do not fully recapitulate the complex physiological conditions of the in vivo tumor microenvironment. Therefore, our data should be considered indicative rather than conclusive. In our future studies, we will incorporate co‐culture systems and 3D cell culture models to gain deeper insights into the interactions between our microreactors and a model that better mimics the complex pancreatic microenvironment.

## 3 Conclusions

This study exploited the LbL methodology to engineer multicatalytic colloidal reactors with controlled membrane architecture at the nano‐ and micrometer scale. The versatility of our approach, which includes the assembly of engineered enzymes (i.e., GOx and HRP) and inorganic nanozymes (i.e., Mn_3_O_4_ nanoparticles) together with synthetic polyelectrolytes, allowed us to optimize the compartmentalization of catalytic units for efficient enzymatic cascade reactions. The synthesized formulations were robust and retained their catalytic activity for up to 5 days at 37 °C, making them compatible with common in vitro studies. Additionally, we explored the possibility of excluding noncatalytic building blocks (i.e., PAH and PSS), resulting in multicatalytic reactors with minimal “ballast” material.

These novel hybrid organic–inorganic microreactors produced hydrogen peroxide in situ, which was subsequently utilized by Mn_3_O_4_ nanozymes for peroxidation reactions. The optimized configuration, with both catalytic units in close proximity, was tested in an in vitro model with human pancreatic cancer cells (MPC2) and stellate cells (hPSCs). This preliminary assessment showed that the microreactors could influence cellular metabolic activity, particularly in the more sensitive hPSCs. As Mn_3_O_4_ nanozymes exhibit both catalase‐ and peroxidase‐like activity, we anticipate that the biological response results from a complex interplay affecting the cellular redox balance. Further investigation is needed to fully elucidate these mechanisms.

Our findings highlight the potential biomedical applications of the developed microreactors, particularly in targeting tumor‐supportive stromal cells within the pancreatic tumor microenvironment. However, the limitations of 2D cell cultures necessitate more advanced biological models. Future studies should clarify the underlying mechanisms, such as lipid peroxidation and oxidative modification of cellular biomolecules, and examine the specific roles of ROS and nanozyme activity. Incorporating 3D tumor spheroids and co‐culture systems, followed by preclinical evaluation, would improve understanding of the microreactors’ biological effects and therapeutic potential. Optimizing enzyme‐to‐nanozyme ratios, scaling up synthesis protocols, and improving stability are essential for clinical translation. This modular, versatile LbL strategy offers promising avenues for biomedical and catalytic applications, including biosensing.

## 4 Experimental Section

4.1

4.1.1

##### Materials

Following reagents were purchased and used without purification: N‐acryoxysuccinimide (NAS, MW 169.13 g mol^−1^, 99%, Scharlab), glucose oxidase from *Aspergillus niger* (GOx, MW 160 kg mol^−1^, E.C. 1.11.1.7, ≤100,000 U g^−1^, Sigma–Aldrich), horseradish peroxidase (HRP, MW 44 kg mol^−1^, E.C. 1.11.1.7, ThermoFisher), potassium phosphate dibasic (MW 174.18 g mol^−1^, ≥98%, Sigma Aldrich), potassium phosphate monobasic (MW 136.09 g mol^−1^, Sigma Aldrich), potassium permanganate (KMnO_4_, MW 158.034 g mol^−1^, ACS 99% min, ThermoFisher Scientific (UK)), poly(allylamine hydrochloride), (PAH, MW 93.56 g mol^−1^, ThermoFisher Scientific (UK)), dimethyl sulfoxide (DMSO, Sigma), acrylamide (AMm, MW 115.13 g mol^−1^, 97%, Sigma Aldrich), monoacryloxyethyl phosphate (MAEP, MW 196.1, 97%, PolySciences), acrylic acid (Aam, MW 72.06 g mol^−1^, >99%, Merck), (3‐Acrylamidopropyl)trimethylammonium chloride (APTAC, MW 206.71 g mol^−1^, 75% wt. in water, Sigma Aldrich), N, N′‐methylenebisacrylamide (BIS, MW 157.17 g mol^−1^, 99%, Sigma–Aldrich), ammonium persulfate (APS, MW 228.20 g mol^−1^, 98%, Sigma–Aldrich), N,N,N’,N’‐tetramethylethylenediamine (TEMED, d 0.775 g mL^−1^, 99%, Sigma–Aldrich), sucrose (MW 342.30 g mol^−1^, 99.7%, Acros Organics), sodium chloride (NaCl, MW 58.44 g mol^−1^, Labkem), PageRuler Plus Prestained Protein Ladder (Biorad), 30% Acrylamide/Bis solution (37.5:1, Biorad), D‐glucose (MW 180.16 g mol^−1^, ≤99.5%, Sigma–Aldrich), 2,2′‐Azino‐bis(3‐ethylbenzothiazoline‐6‐sulfonic acid) diammonium salt (ABTS, MW 514.62 g mol^−1^, ThermoFisher), Tris(hydroxymethyl)aminemethane (Tris, MW 121.14 g mol^−1^, Scharlab), calcium chloride (CaCl_2_, MW 110.98 g mol^−1^, ≥97%, Sigma–Aldrich), sodium carbonate Na_2_CO_3_ (MW 105.99 g mol^−1^, Scharlab), poly(sodium‐p‐styrenesulfonate) (PSS, MW 70,000 g mol^−1^, Acros Organics), poly(allylamine hydrochloride) (PAH, MW 17,500 g mol^−1^, ThermoFisher), 3,5‐dinitrosalicylic acid (DNS, MW 228.12 g mol^−1^, 98%, Merck), phenol (MW 94.11 g mol^−1^, Sigma–Aldrich), sodium sulfite (MW 126.4 g mol^−1^, 98%, abcr), sodium hydroxide (NaOH, 40.0 g mol^−1^, 98%, Sigma–Aldrich), hydrogen peroxide (H_2_O_2_, MW 34.01 g mol^−1^, 30% wt, Merck), sodium acetate (AcONa, MW 82.03 g mol^−1^, Scharlab), and Amplex red (Fisher). In addition, filter membranes (30 kDa MWCO, AmiconUltra, 15 mL) and dialysis membranes (10 kDa MWCO, SnakeSkin, ThermoFisher) were used for purification.

##### Dynamic Light Scattering and ζ‐Potential

Dynamic light scattering and ζ‐potential were carried out on a Malvern Zetasizer Nano (Malvern Panalytical) at room temperature. The presented results are calculated as the means of three consecutive measurements. Proteins (refractive index = 1.45) and single enzyme nanogels (SENs) were diluted at 1 mg mL^−1^ in PBS for size measurements. The hydrodynamic size and polydispersity of Mn_3_O_4_ nanoparticles were determined in MilliQ water at a concentration of 100 μg mL^−1^. For ζ‐potential determination, proteins and SENs were diluted at 0.5 mg mL^−1^ in KCl (10 mM). The assembly of building blocks (i.e., polyelectrolytes, proteins, Mn_3_O_4_ nanoparticles, SENs) on CaCO_3_‐based microparticles or mesoporous silica nanoparticles was also monitored via ζ‐potential measurements at a concentration of 0.3 mg mL^−1^ in NaCl (5 mM).

##### Ultraviolet‐Visible Spectroscopy

Measurements were performed using a Synergy Neo2 Multimode Reader (BioTek). Spectra were acquired from 240 to 900 nm with a step size of 1 nm. Measurements were performed in 96‐well plates, with a total sample volume of 200 μL.

##### Sodium Dodecyl Sulfate Polyacrylamide Gel Electrophoresis (SDS‐PAGE)

Characterization was performed in a Mini‐PROTEAN Tetra System (BioRad) cuvette connected to a PowerPac Basic (BioRad) power source. Before loading to 12% acrylamide gels, the samples (18 μL, 5 μg) were mixed with the loading buffer (2 μL) and denatured at 95 °C for 5 min. The samples were run for 2 h at a constant voltage of 90 V. The gels were subsequently stained with Coomassie Blue for 15 min and destained overnight in water. Gels were imaged at a GelDoc Go Imaging System (BioRad).

##### Scanning Electron Microscopy (SEM)

The morphology of the multilayer nano‐ and microparticles was evaluated using SEM (Hitachi FEG‐SEM S‐4800) with an acceleration voltage of 5.0 kV. The samples were coated with a 10 nm layer of gold using an Emitech K550X ion sputter.

##### Transmission Electron Microscopy (TEM)

The morphological characterization of the Mn_3_O_4_ nanoparticles was performed using bright‐field transmission electron microscopy with a 120 kV Talos L120C microscope. Sample preparation involved casting 20 μL of the nanoparticle dispersion (100 μg mL^−1^) onto 300 mesh copper grids with carbon film coating and allowing it to rest for 5 min. Afterward, the excess liquid was gently removed using filter paper. The samples were left to dry completely for several hours before imaging.

##### Attenuated Total Reflectance Fourier‐Transform Infrared (ATR‐FTIR)

Samples were drop‐cast on silicon wafers and dried at 800 mbar. This process was repeated five times with drops of 4 μL to obtain well‐defined and thick layers of the sample. The sample spectra were subsequently measured with a Nicolet 6700‐Golden Gate MKII device spectrophotometer with an attenuated total reflectance (ATR) sampling stage and a diamond glass. All spectra were measured from 600 to 4000 cm^−1^, with a resolution of 4 cm^−1^.

##### X‐Ray Diffraction (XRD)

The synthesized Mn_3_O_4_ nanoparticles were subjected to an XRD analysis using a PHILIPS X’PERT PRO automatic diffractometer operating at 40 kV and 40 mA, in theta‐theta configuration, a secondary monochromator with Cu‐Kα radiation (λ = 1.5418 Å), and a PIXcel solid‐state detector.

##### X‐Ray Photoelectron Spectroscopy (XPS)

XPS was performed using an ESCA2SR spectrometer (ScientaOmicron GmbH) using monochromatic Al Kα radiation (1486.6 eV; 20 mA emission at 300 W; 1 mm spot size) with a base vacuum pressure of 1 × 10^−9^ mbar. Charge neutralization was achieved using a low‐energy electron flood source (FS40A, PreVac). Survey spectra were measured using 150 eV pass energy and core levels with 50 eV pass energy. Binding energy (BE) scale calibration was performed using C—C in the C 1s photoelectron peak at 284.8 eV. Analysis and curve fitting were performed using Voigt‐approximation peaks using CasaXPS (N. Fairley, CasaXPS, 2019). https://www.casaxps.com. Ultraviolet photoelectron spectroscopy (UPS) was performed using a He lamp (He I, 21.2 eV, FOCUS GmbH, 100 mA emission at 70 W) and an analyzer pass energy of 5 eV, with the sample biased by 20.0 V.

##### Synthesis of the Single Enzyme Nanogels

The synthesis of SENs is based on the group's previous work^[^ ^29^, ^31^ ^]^ and consists of two steps.

##### Acryloylation of Enzymes

Before synthesizing the SENs, commercial GOx and HRP were pre‐activated by modifying their accessible lysine residues with NAS. The selected enzymes were conditioned in a phosphate buffer with high ionic strength (300 mM, pH 8.0). NAS was dissolved in DMSO (50 mg mL^−1^, 20 eq.) and slowly added to the protein solutions (4 mg mL^−1^, 5 mL, 1 eq.). The solutions were shaken for 2 h at room temperature. The reactions were stopped by overnight dialysis against 2 L of sodium phosphate buffer (15 mM, pH 8.0) using 10 kDa MWCO membranes. Finally, the acryloylated enzymes were concentrated to 25 mg mL^−^^1^ using Amicon filters (30 kDa MWCO).

##### Radical Polymerization and In Situ Encapsulation

The previously acryloylated GOx and HRP were mixed with selected monomers (HEEA, AMm, MAEP, AAm, APTAC), crosslinker (BIS), and the initiator/catalyst (APS/TEMED) mixture in the molar ratios summarized in Table S2 (see Supporting Information). The monomers were optimized to introduce the desired motifs (positive or negative charges) to the nanogels and to ensure a good protein encapsulation while maintaining the polymeric mantle's thickness.^[^ ^31^ ^]^ For the synthesis of the GOx@APTAC, GOx@MAEP, and HRP@APTAC SENs, the protein concentrations in the reaction were 12, 30, and 30 μM, respectively, with a final reaction volume of 5.208 mL in sodium phosphate buffer (30 mM, pH 8.0). In the case of GOx@AA SENs, the protein concentration was set at 20 μM, using a final volume of 0.937 mL of sodium phosphate buffer (30 mM, pH 6.0). Sucrose (5%, w/v) was added to both reactions to enhance the interaction between the monomers and the proteins. DMSO (15%, v/v) was included in the reactions to address the insolubility of BIS in water. The reaction mixture was deoxygenated by continuous bubbling of argon. Finally, the reaction was initiated by adding a 10% (w/v) solution of APS and TEMED. The reactions were carried out for 2 h at room temperature with vigorous stirring. Any unreacted monomers were removed by dialysis using a 10 kDa MWCO membrane against sodium chloride (100 mM) and sodium phosphate buffer (15 mM pH 6.0 or pH 8.0 for GOx@AA and GOx@APTAC, GOx@MAEP and HRP@APTAC, respectively). Samples were concentrated using Amicon filters (30 kDa MWCO).

##### Synthesis of CaCO_3_ Template

For the synthesis of CaCO_3_ microparticles, a previously reported procedure was followed.^[^ ^50^ ^]^ Briefly, an aqueous solution of CaCl_2_ (0.33 mL, 1 M) was mixed with either distilled water (0.33 mL) to fabricate the empty template or with a solution containing the enzyme of interest (i.e., GOx) in Tris Buffer (0.33 mL, 0.05 M) at pH  7.0 to fabricate the GOx‐loaded CaCO_3_ microparticles. Subsequently, a Na_2_CO_3_ solution (0.33 mL, 1 M) was added, and the mixture was stirred vigorously for 30 s to allow the formation of CaCO_3_ microparticles. After 15 min of incubation, the particles were collected by centrifugation and washed with NaCl 5 mM to remove unreacted salts. As a result of this synthesis procedure, ≈30 mg of CaCO_3_ microparticles were obtained. Alternatively, mesoporous silica nanoparticles (particle size = 200 nm; pore size = 4 nm; Sigma–Aldrich) were used as a template for some of the formulations.

##### Assembly of the Building Blocks

In the present study, several building blocks (i.e., polyelectrolytes, SENs, and Mn_3_O_4_ nanoparticles) have been deposited on the surface of CaCO_3_ microparticles or mesoporous silica nanoparticles by electrostatic interactions following the LbL approach. In the case of polyelectrolytes (i.e., PAH or PSS), the particles (10 mg mL^−1^ in the case of CaCO_3_ microparticles and 5 mg mL^−1^ in the case of mesoporous silica nanoparticles) were incubated in an aqueous solution containing the polyelectrolyte (2 mg mL^−1^ in 500 mM NaCl solution at pH = 6.5) for 15 min under shaking.

Several concentrations of SENs were considered for the assembly of SENs during this incubation step. Unless otherwise stated, the particles (10 mg mL^−1^ in the case of CaCO_3_ microparticles and 5 mg mL^−1^ in the case of mesoporous silica nanoparticles) were incubated with the solution of SENs (e.g., GOx@APTAC, GOx@AA, GOx@MAEP, or HRP@APTAC) at a concentration of 0.1 mg mL^−1^ in Tris Buffer (10 mM, pH  7.0) under shaking.

For the assembly of Mn_3_O_4_ nanoparticles, the particles (10 mg mL^−1^ of CaCO_3_ microparticles) were incubated in a solution containing PAH (2 mg mL^−1^) and Mn_3_O_4_ nanoparticles (4 mg mL^−1^) in Tris Buffer (10 mM, pH = 7) under stirring. The absence of PAH in this step resulted in the flocculation of Mn_3_O_4_ nanoparticles and the unsuccessful assembly during the LbL approach. Finally, a last formulation containing the Mn_3_O_4_ nanoparticles and GOx@APTAC was also considered. Here, the particles (10 mg mL^−1^ of CaCO_3_ microparticles) were co‐incubated with PAH (2 mg mL^−1^), GOx@APTAC (0.1 mg mL^−1^), and Mn_3_O_4_ (4 mg mL^−1^) in Tris Buffer (10 mM, pH  7.0) under shaking. After each deposition step, the particles were collected by centrifugation and washed with NaCl (5 mM) to remove non‐assembled building blocks. The successful assembly of the building blocks was assessed by ζ‐Potential measurements.

##### Synthesis of Mn_3_O_4_ Nanoparticles

Manganese (II, III) oxide (Mn_3_O_4_) nanoparticles were synthesized following a multi‐step protocol. Initially, Solution A was prepared by dissolving 163 mg of potassium permanganate (KMnO_4_) in 37.5 mL of MilliQ water and stirring at 300–500 rpm for 10 min. Separately, Solution B was prepared by dissolving 233.75 mg of PAH in 12.5 mL of MilliQ water, also stirred at 300–500 rpm for 10 min. The two solutions were then combined and stirred at 400 rpm for 5 min to form dispersion C. The resulting dispersion was concentrated using Vivaspin 20 centrifugal concentrator (MWCO: 50 kDa) by centrifugation at 4000 rpm (3800 g) for 30–60 min (Thermo Fisher Sorvall legend X1 equipped with a Thermo Scientific TX‐400 4 × 400 mL swinging bucket rotor) or until its volume reached 10 mL. If the dispersion volume was lower, additional MilliQ water was added to increase to 10 mL. The apical dispersion was transferred to 15 mL Falcon tubes, and tip‐sonicated at an amplitude of 15 micron for 5 min (MSE Soniprep 150, 23 KHz) on ice before being transferred into ultracentrifuge tubes (1 mL each). Ultracentrifugation was performed at 50,000 rpm for 30 min (Beckman Coulter Optima MAX, rotor: TLA‐120.2 Fixed Angle 30°), and the sediment was discarded while retaining the supernatant. The supernatant was centrifuged again at 120,000 rpm (627,000 g) for 30 min. At this stage, the precipitate was collected and redispersed in MilliQ water using a tip‐sonicator (30 s to 1 min at 15 μm amplitude for each small tube) while ensuring the precipitate was carefully detached from the tube walls using a spatula before sonication. The redispersed samples from the small ultracentrifuge tubes were combined in a 50 mL Falcon tube and sonicated at an amplitude of 10 microns in an ice bath for 10 min before being transferred back to ultracentrifuge tubes. This process of centrifugation at 120,000 rpm, collection, and redispersion was repeated twice to ensure the removal of noncoated PAH and very small particles. After the final centrifugation, the precipitate was collected from each ultracentrifuge vial with the help of a spatula and a tip sonicator at 15 microns amplitude for 30 s and transferred to a 15 mL Falcon tube. The resulting dispersion was filled with MilliQ water until it reached 10 mL and was then sonicated at an amplitude of 10 microns for 15 min in an ice bath. Finally, the sample was filtered using a 0.22 μm PVDF filter, ensuring that no more than 3.5 mL was filtered at a time to avoid blockage. The filtrate was transferred to 15 mL Falcon tubes, and an aliquot was taken for freeze‐drying and concentration calculation. The Mn_3_O_4_ nanoparticles were stored at 4 °C until analysis.

##### Cell Lines and In Vitro Experiments

MiaPaCa‐2 (MPC2) and human pancreatic stellate cells (hPSCs) were cultured in Dulbecco's Modified Eagle Medium (DMEM, Sigma–Aldrich, D6429) containing sodium bicarbonate, L‐glutamine, and sodium pyruvate, supplemented with 10% fetal bovine serum (FBS) and 1% penicillin‐streptomycin. The cells were maintained at 37 °C in a humidified atmosphere with 5% CO_2_, and the medium was refreshed every 2–3 days. Both cell lines were passaged upon reaching 80%–90% confluency using trypsin‐EDTA solution, with cells used for experiments limited to passages 3–10 to ensure consistency.

To assess metabolic activity, 5000 cells per well (15,625 cells cm^−^^2^) were seeded into 96‐well plates containing 200 μL of culture medium and incubated at 37 °C with 5% CO_2_ for 24 h. Following this incubation, each well received 10 or 20 μL of phosphate‐buffered saline (PBS) dispersions containing LbL‐GOx, LbL‐Mn, or LbL‐Mn‐GOx particles to achieve final concentrations of 1, 5, and 10 μg mL^−1^, respectively. Control wells were treated with the highest volume of PBS alone to ensure that the vehicle did not affect cell viability. At predetermined time points of 24 and 72 h, the culture medium was carefully removed from each well, and the cells were gently washed once with 1X PBS to eliminate any residual particles. Subsequently, 100 μL of a working solution—prepared by mixing equal volumes of 1X PBS and CellTiter‐Glo Reagent—was added to each well. The plates were then placed on a vertical shaker and agitated in the dark at 600–700 rpm to facilitate cell lysis and ensure thorough mixing. After incubation, the luminescent signal was stabilized by allowing the plates to rest at room temperature for 10 min. The contents of each well were then transferred to white 96‐well plates with opaque bottoms, and luminescence was measured using a BioTek Synergy 2 plate reader. The luminescence readings were normalized to the average values of the corresponding control samples at each respective time point to account for any background signal and to facilitate comparative analysis.

##### Statistical Analysis

Continuous variables are reported as mean ± standard deviation (SD). Analyses were conducted exclusively on biological experiments. Group comparisons were evaluated using one‐way analysis of variance (ANOVA), followed by Tukey's post hoc test for data meeting assumptions of normality and homogeneity of variances. Normality was assessed in GraphPad Prism version 10 using the D’Agostino and Pearson, Anderson–Darling, Shapiro–Wilk, and Kolmogorov–Smirnov tests. Most datasets passed the Shapiro–Wilk and D’Agostino and Pearson tests (*p *> 0.05), with no consistent violations across all tests. Given the balanced design (*n* = 9 per group; *N* = 3 independent experiments), ANOVA was considered appropriate due to its robustness to minor deviations from normality. A *p* value ≤ 0.05 was considered statistically significant. N denotes the number of independent experiments, and *n* refers to technical replicates.

## Conflict of Interest

The authors declare no conflict of interest.

## Author Contributions

**Aitor Ontoria**: conceptualization, methodology, investigation, data curation, and writing—original draft. **Irene Alonso-Sampedro**: investigation, formal analysis, and data curation. **Yixuan Yan**: investigation and writing—review and editing. **Ayşe Latif**: investigation and writing—review and editing. **Ben F. Spencer**: data curation, formal analysis, and writing—review and editing. **Aitor Larrañaga**: conceptualization, supervision, writing—review and editing, and funding acquisition. **Ana Beloqui**: conceptualization, supervision, writing—review and editing, and funding acquisition. **Christos Tapeinos**: conceptualization, supervision, writing—review and editing, and funding acquisition.

## Supporting information

Supplementary Material

## Data Availability

The data supporting this study's findings are available from the corresponding authors upon reasonable request.
