# Supplementary material for: Immobilization of Enzyme–Polymer Hybrids and Nanozymes Through Electrostatic Interactions: Toward Multicatalytic Microreactors with Controlled Nanoarchitecture
Source: Small Sci. 2025 Jun 10;5(8):2500167. doi: 10.1002/smsc.202500167 (PMC12362757; doi:10.1002/smsc.202500167)
Supplement: Supplementary file 1 — Supplementary Material [file SMSC-5-2500167-s001.pdf]

Supporting Information

Immobilization of enzyme-polymer hybrids and nanozymes through electrostatic interactions: towards multi-catalytic microreactors with controlled nano-architecture

Aitor Ontoria, Irene Alonso-Sampedro, Yixuan Yan, Ayşe Latif, Ben F. Spencer, Aitor Larrañaga, Ana Beloqui,** Christos Tapeinos*

1. METHODS

1.1. Immobilization of free proteins in multilayer particles through electrostatic interactions

In this experiment, we aimed to assess the feasibility of immobilizing two model enzymes, GOx and HRP, through electrostatic interactions in the layers of the multilayered particles used in this study. For that, $CaCO_3$ microparticles with an exposed layer of PAH were prepared (synthesis details are collected below in Section 3.4). Free protein solutions (0.1 mg mL^{-1}) were mixed with PAH-coated $CaCO_3$ microparticles following the same procedure as that used afterward with the protein hybrids (description in Section 3.4). To assess the immobilization efficiency, we measured the protein immobilization yield (description in Section 3.5) and the ζ-potential before and after the procedure (description in Section 2.1). The obtained results are presented in **Table S1**.

Table S1. Immobilization yield and ζ-potential of the particles upon free proteins and SENs deposition. ζ-potential measured for free enzymes and SENs is also included as a reference.

**Immobilization yield (%)**	
PAH-coated $CaCO_3$ + HRP	2.2
PAH-coated $CaCO_3$ + GOx	17.0
PAH-coated $CaCO_3$ + GOx@MAEP	100.0
PSS-coated $CaCO_3$ + HRP@APTAC	100.0
PAH-coated $CaCO_3$ + GOx@AA	100.0
HRP@APTAC-coated $CaCO_3$ + GOx@MAEP	100.0
GOx@MAEP-coated $CaCO_3$ + HRP@APTAC	100.0
PAH-coated SiO_2 + GOx@MAEP	100.0
PSS-coated SiO_2 + HRP@APTAC	100.0
**ζ-potential (mV)**	
HRP	-11.4 ± 1.2
GOx	-14.6 ± 0.5
HRP@APTAC	+7.4±0.8
GOx@MAEP	-23.6±1.7
GOx@AA	-33.3±1.6
GOx@APTAC	+27.6 ± 0.9
PHA-coated $CaCO_3$	+12.3 ± 2.4

PHA-coated $CaCO_3$ + HRP	+3.4 ± 1.1
PHA-coated $CaCO_3$ + GOx	+7.9 ± 0.5

These experiments demonstrated that HRP and GOx cannot effectively immobilize on PAH-coated $CaCO_3$ microparticles through solely electrostatic interactions.

1.2. Synthesis of single enzyme nanogels (SENs)

Table S2. Synthesis conditions utilized in this work to fabricate SENs, with all values expressed as equivalents of monomers, APS, and TEMED relative to GOx.

Code	[1]HEAA	[1]AMm	[2]BIS	[1]MAEP	[3]AAm	[1]APTAC	[1]APS	[3]TEMED	[4]Vf (mL)
GOx@AA	-	2000	600	-	1000	-	400	200	0.937
GOx@APTAC	-	2000	600	-	-	3000	900	450	5.208
GOx@MAEP	600		600	1000	-	-	600	300	5.208
HRP@APTAC	600		400	-	-	2000	400	200	5.208

[1]Stock solution of 10% (w/v) in MilliQ water. [2]Stock solution of 10% (w/v) in DMSO. [3]No dilution. [4]Final reaction volume. Buffers used for the reaction are phosphate buffer (PB) 30 mM, pH 8.0, and PB 30 mM, pH 6.0 for GOx@APTAC and GOx@AA, respectively.

Table S3. The specific activity of the different enzyme hybrids.

Sample	Specific activity (U mg^{-1})
**aGOx**	118.4±3.7
**GOx@APTAC**	66.6±4.7
**GOx@AA**	82.9±16
**GOx@MAEP**	18.8±0.5
**Free HRP**	398.3±249.1
**HRP@APTAC**	354.1±106.05

1.3. Characterization of single enzyme nanogels (SENs)

SDS-PAGE. The encapsulation yield of the synthesized SENs was characterized by SDS-PAGE. In this technique, the electrophoretic mobility of the samples varies depending on the size of the sample. While the free protein migrates until the 80 kDa band of the ladder, the SENs stay at the top part of the lane. This difference in migration is an indication of the successful encapsulation of the enzyme in the nanogels. The relation in intensity of the different bands indicates the yield of encapsulation of the different SENs. The considered SENs were satisfactorily synthesized according to their electrophoretic mobility.

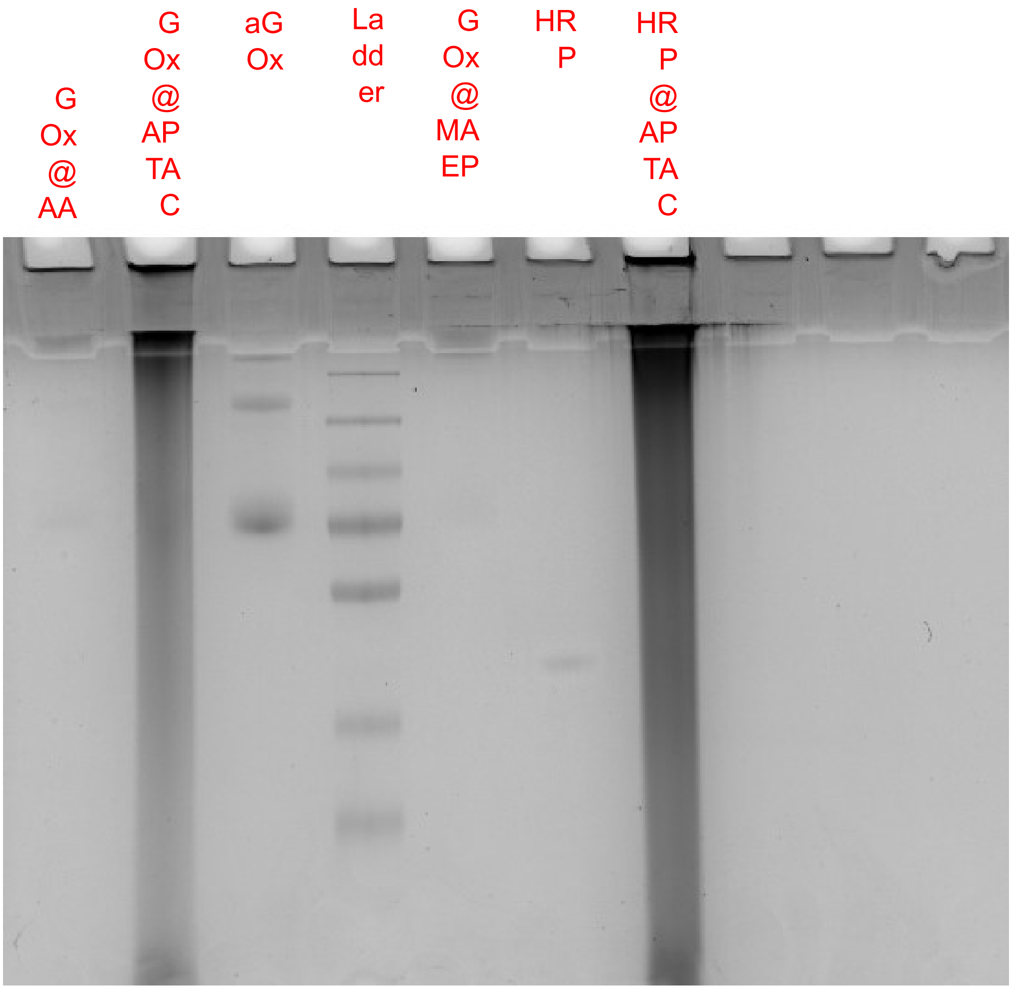

Figure S1. SDS-PAGE of the samples. From left to right, GOx@AA, GOx@APTAC, aGOx, MW ladder, GOx@MAEP, HRP and HRP@APTAC.

DLS. Sample size distribution was measured by DLS. All samples exhibited a increased hydrodynamic diameter upon encapsulation. The hydrodynamic diameter (mean by number) of the free acryloylated GOx (aGOx) was 6.9±0.3nm and the polydispersity index PDI was 0.201±0.042. For the GOx-based SENs, the hydrodynamic diameters were 8.9±1.4 nm (PDI 0.274±0.012), 9.2±1.1 nm (PDI 0.399 ±0.0118), 7.5±0.5 nm (PDI 0.267±0.001) for GOx@APTAC, GOx@AA and GOx@MAEP, respectively. The hydrodynamic diameter of free HRP was 5.6±0.1 nm and the PDI 0.552±0.137, whereas the HRP@APTAC hybrid exhibited a hydrodynamic diameter of 6.7±0.4 nm (PDI 0.438±0.008).

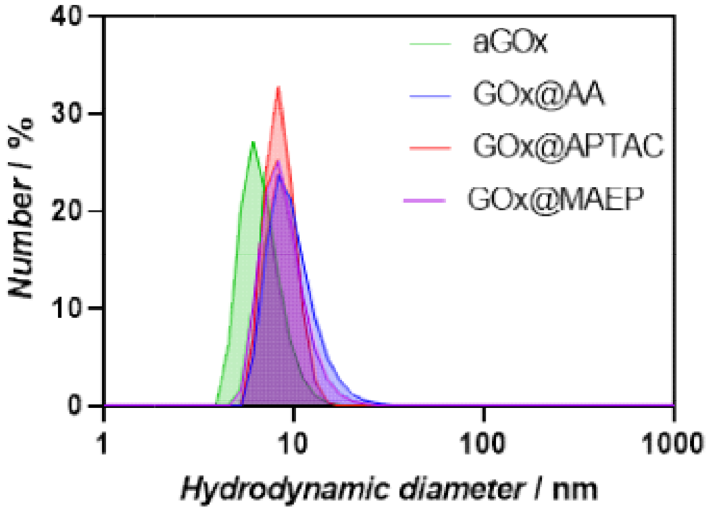

Figure S2. Hydrodynamic diameter measurements by dynamic light scattering of the formulated GOx samples. Measurements were made in triplicate.

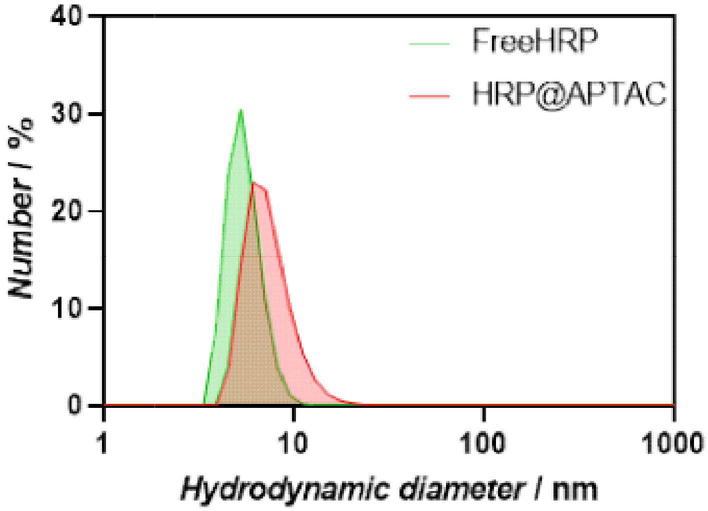

Figure S3. Hydrodynamic diameter measurements by dynamic light scattering of the formulated HRP samples. Measurements were made in triplicate.

FT-IR. FT-IR spectra of the synthesized SENs showed significant differences. Samples containing APTAC as monomer exhibited a characteristic peak of this monomer at 967 cm^{-1} corresponding to the -N-$(CH_3)_3$ bending. [1] This peak suggests the incorporation of the monomer within the polymeric mantle. Moreover, these spectra showed a broadening of the peak at the 3000-3300 cm^{-1} region. In the sample containing MAEP as a monomer, a peak can be observed at 1725 cm^{-1}. This peak can be assigned to the symmetric stretching vibration of the carbonyl group of MAEP. Finally, in the sample containing AAm as a charge donating monomer, a peak at 1397 cm^{-1} can be observed. This peak corresponds to the O-H bend. Also, in this sample, a peak can be discerned at 1710 cm^{-1}, which corresponds to the C=O stretching.

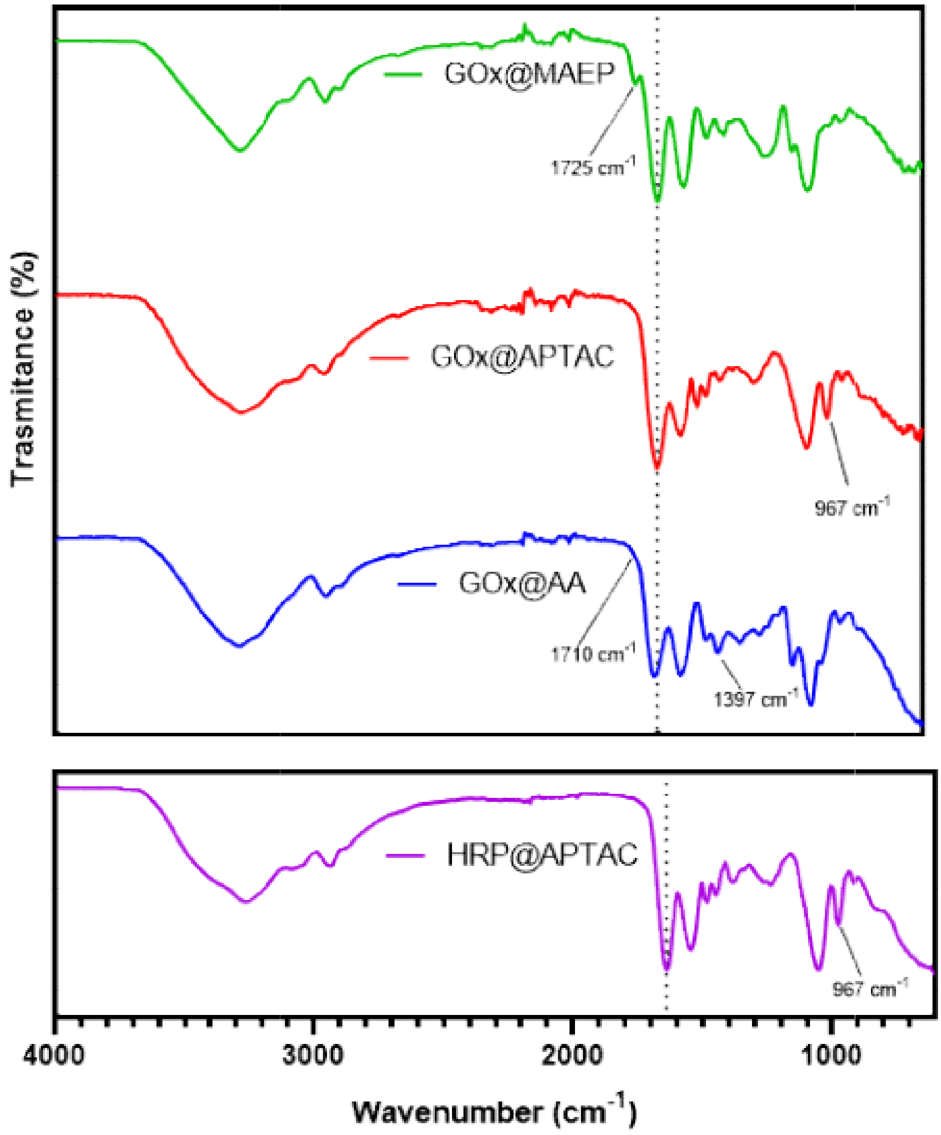

Figure S4. FTIR spectra of the different synthesized SENs.

Catalytic performance of the SENs. The catalytic performance of the synthesized SENs was assessed by monitoring spectroscopically the ABTS ($\varepsilon_{ABTS,418}$= 36000 M^{-1} cm^{-1}) oxidation at 418 nm. For GOx, both acryloylated enzyme and SENs (0.001 mg mL^{-1}) were mixed in Tris·HCl (10 mM, pH 7.0) with glucose (25 mM), ABTS (1 mM) and HRP (0.0349 mg mL^{-1}). As for HRP, both acryloylated enzyme and SENs (0.001 mg mL^{-1}) were mixed in Tris·HCl (10 mM, pH 7.0) with ABTS (1 mM). Reactions were carried out while shaking at room temperature, and absorption at 418 nm was monitored over time. All samples exhibited a decrease in the specific activity compared to the free acryloylated protein (see Figure and Table below). This decrease in activity can be attributed to the presence of the crosslinked polymeric mantle around the protein, which can limit the diffusion of substrates from the bulk of the solution to the active site of the protein.

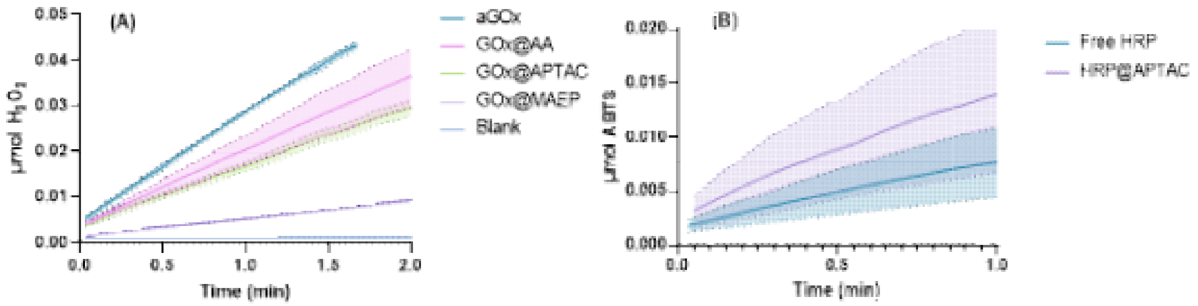

Figure S5. Kinetic monitoring of the samples. Absorption at 418 nm was tracked over time of a solution containing (A) 0.001 mg mL^{-1} of GOx, 25 mM glucose, 1 mM ABTS and HRP in tris·HCl 10 mM pH 7.0 and (B) 0.0001 mg mL^{-1} of HRP, and 0.27 mM ABTS in Citrate buffer 50 mM pH 5.0

1.4. Fabrication of multilayer nano- and microreactors

The procedure detailed in the main manuscript allowed us to fabricate several formulations detailed along the main manuscript. The CaCO3 template was eliminated by incubating the samples with EDTA at concentrations ranging from 60 to 100 mM for the fabrication of hollow capsules.

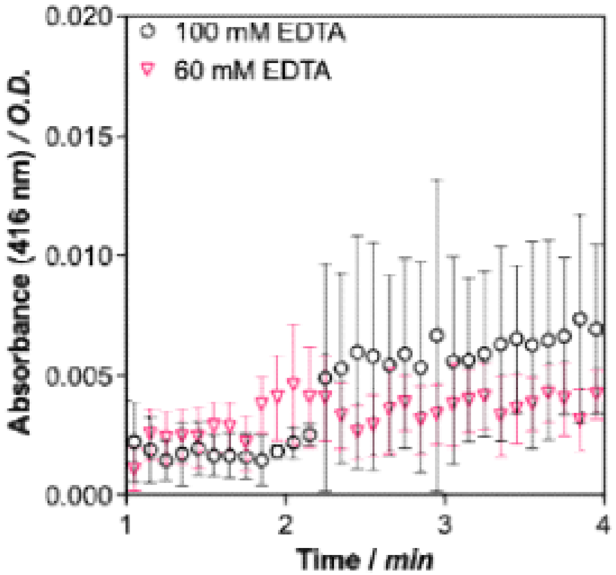

Figure S6. Kinetic measurements performed for the microcapsules.

1.5. Characterization of multilayer nano- and microreactors

Quantification of immobilized GOx@MAEP and HRP@APTAC. The immobilization of GOx@MAEP and HRP@APTAC was calculated by the Bradford assay. Briefly, 150 µL of the sample were mixed with 150 µL of Bradford reactant in a 96-well plate. Samples were incubated at room temperature for 10 min, and the UV absorbance at 595 nm was subsequently measured. Concentration was calculated by interpolating the sample in a calibration curve with the free enzyme.

Scanning electron microscopy (SEM) was used to characterize freshly prepared samples and upon incubation at 37 ºC.

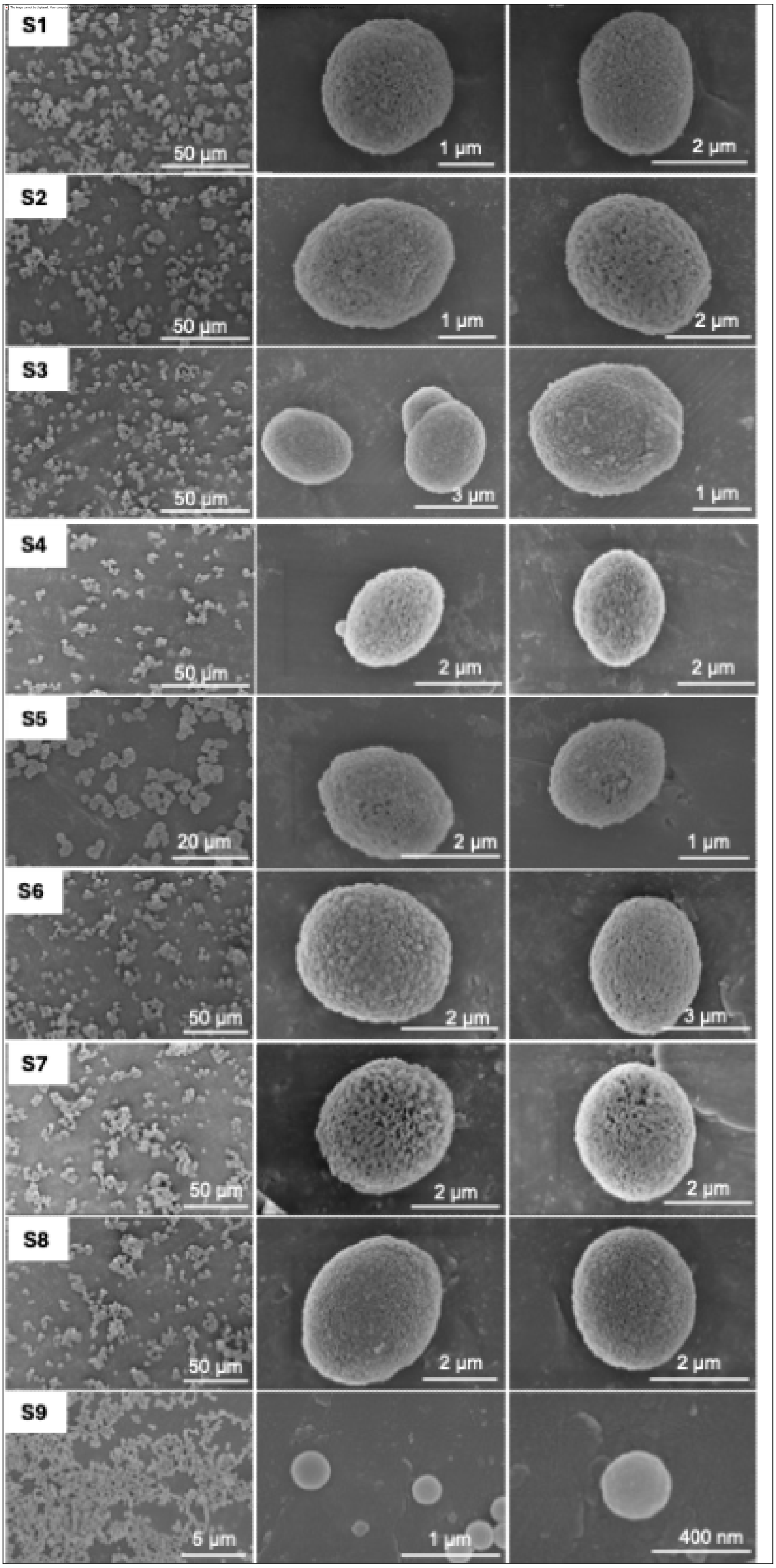
S1
50 µm
1 µm
2 µm
S2
50 µm
1 µm
2 µm
S3
50 µm
3 µm
1 µm
S4
50 µm
2 µm
2 µm
S5
20 µm
2 µm
1 µm
S6
50 µm
2 µm
3 µm
S7
50 µm
2 µm
2 µm
S8
50 µm
2 µm
2 µm
S9
5 µm
1 µm
400 nm

Figure S7. SEM micrographs of freshly prepared samples.

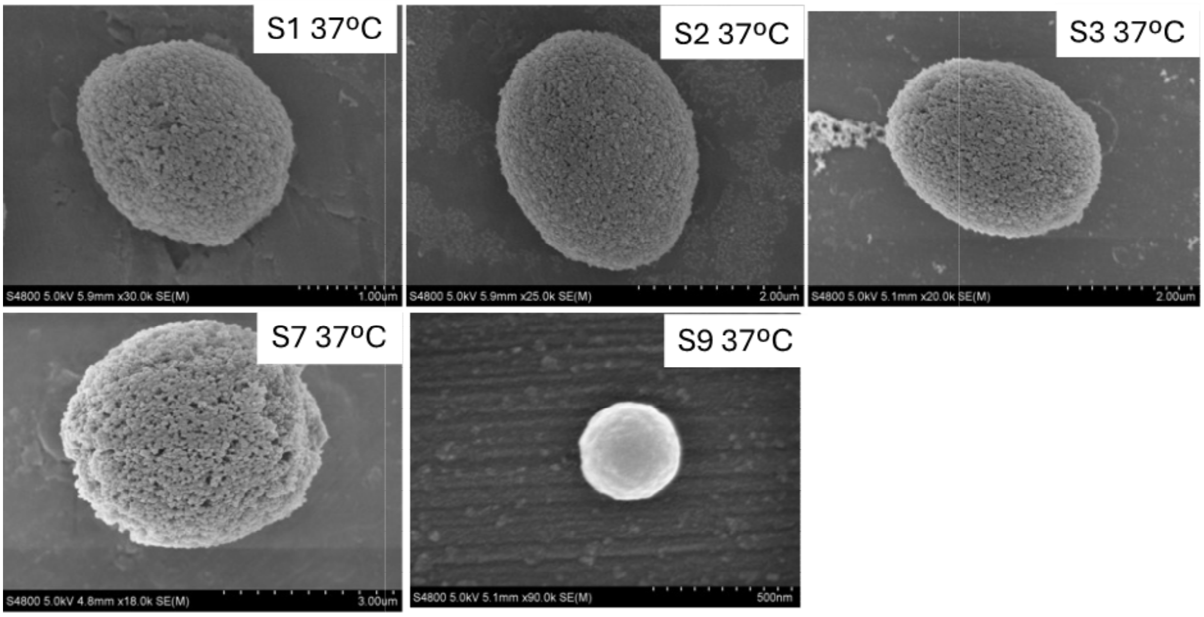

Figure S8. SEM pictographs of samples after 5 days of incubation at 37ºC

1.6. Characterization of Mn_3O_4 nanoparticles

XRD. The XRD spectrum of Mn_3O_4 nanoparticles demonstrates distinct and well-defined diffraction peaks corresponding to the characteristic crystal planes listed in **Table S4a**. These peaks confirm the presence of Mn_3O_4, with prominent reflections. The sharp and intense nature of the peaks reflects the high crystallinity and phase purity of the synthesized material.

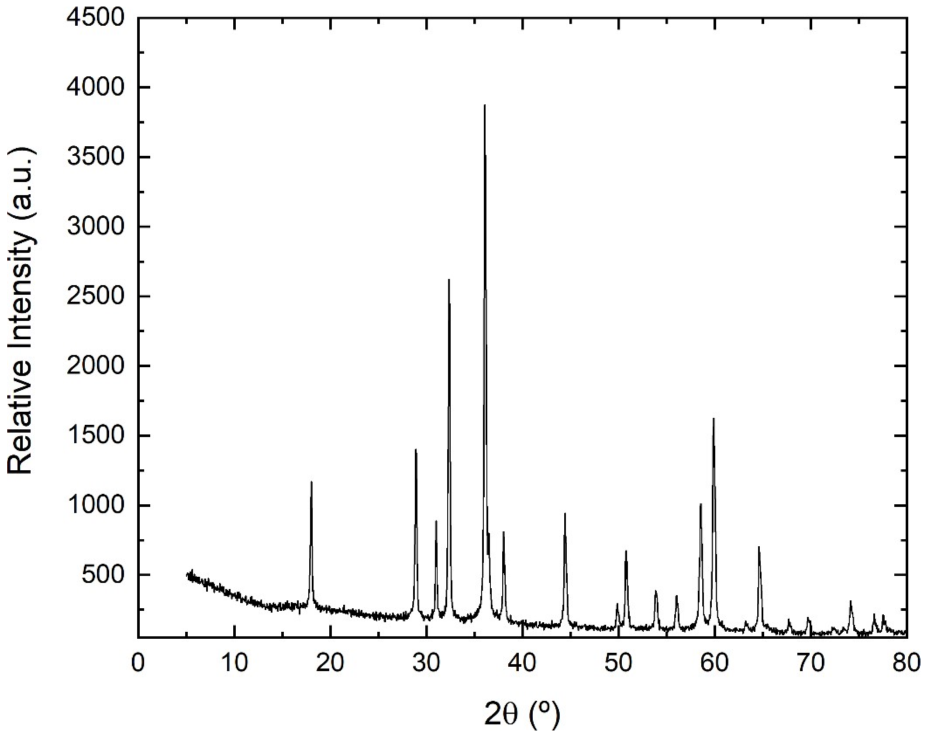

Figure S9. X-Ray diffraction pattern showing the various peaks corresponding to the Mn_3O_4 nanoparticles. The assignment and detailed information for each peak can be found in Table S4a.

Table S4a. Peak list and Miller indices (hkl) for Mn_3O_4 nanoparticles.

Pos. [2θ (deg)]	Height [cts]	FWHM Left [2θ (deg)]	d-spacing [Å]	Rel. Int. [%]	Matched by	Miller Indices (hkl)
18.0147	879.78	0.1535	4.92420	24.07	01-080-0382; 00-044-0141	(101)
28.9075	1207.06	0.1919	3.08871	33.02	01-080-0382; 00-044-0141; 00-050-0866	(112)
31.0083	634.75	0.1919	2.88407	17.36	01-080-0382	(200)
32.3590	2425.77	0.1535	2.76671	66.35	01-080-0382	(103)
36.1046	3655.78	0.1919	2.48781	100.00	01-080-0382	(211)
36.5091	613.01	0.1151	2.46117	16.77	01-080-0382; 00-042-1169; 00-042-1316; 00-044-0141	(004)
38.0409	617.83	0.1535	2.36553	16.90	01-080-0382; 00-042-1316	(202)
44.4209	806.27	0.2686	2.03946	22.05	01-080-0382; 00-042-1169	(204)
49.8555	171.01	0.1919	1.82914	4.68	01-080-0382; 00-042-1316; 00-044-0141	(220)
50.7604	547.06	0.2686	1.79864	14.96	01-080-0382	(105)
53.8565	266.10	0.2686	1.70232	7.28	01-080-0382; 00-042-1316	(312)
56.0176	235.56	0.1919	1.64166	6.44	01-080-0382; 00-030-0820	(303)
58.5126	871.44	0.2686	1.57747	23.84	01-080-0382; 00-042-1316	(224)
59.8683	1459.41	0.1919	1.54495	39.92	01-080-0382; 00-050-0866	(314)
63.2790	55.23	0.2303	1.46965	1.51	01-080-0382	(400)
64.6149	590.00	0.1919	1.44245	16.14	01-080-0382; 00-042-1316	(323)
67.7712	67.85	0.2303	1.38276	1.86	01-080-0382; 00-044-0141	(332)

69.7887	97.69	0.3838	1.34764	2.67	01-080-0382; 00-044-0141	(206)
74.1553	231.93	0.1151	1.27872	6.34	01-080-0382	(411)
76.6011	100.62	0.2686	1.24388	2.75	01-080-0382	(424)
77.5216	118.03	0.1535	1.23139	3.23	01-080-0382	(505)

Table S4b. Identified pattern list, peaks, relative intensities and miller indices for the identified crystal phase of Mn_3O_4 (according to JCPDS database card no. 01-080-0382 and RUFF database card no. R040090 (*https://rruff.info/hausmannite/display=default/R040090*).

Ref. Code	Score	Compound Name	Displacement [2θ (deg)]	Scale Factor	Chemical Formula
01-080-0382	91	Manganese Oxide	-0.036	0.719	Mn_3O_4

2θ (°)	d-spacing (Å)	hkl
18.0	4.92	(101)
28.9	3.09	(112)
32.3	2.77	(103)
36.0	2.49	(211)
44.4	2.04	(220)
58.5	1.58	(224)
64.7	1.44	(400)

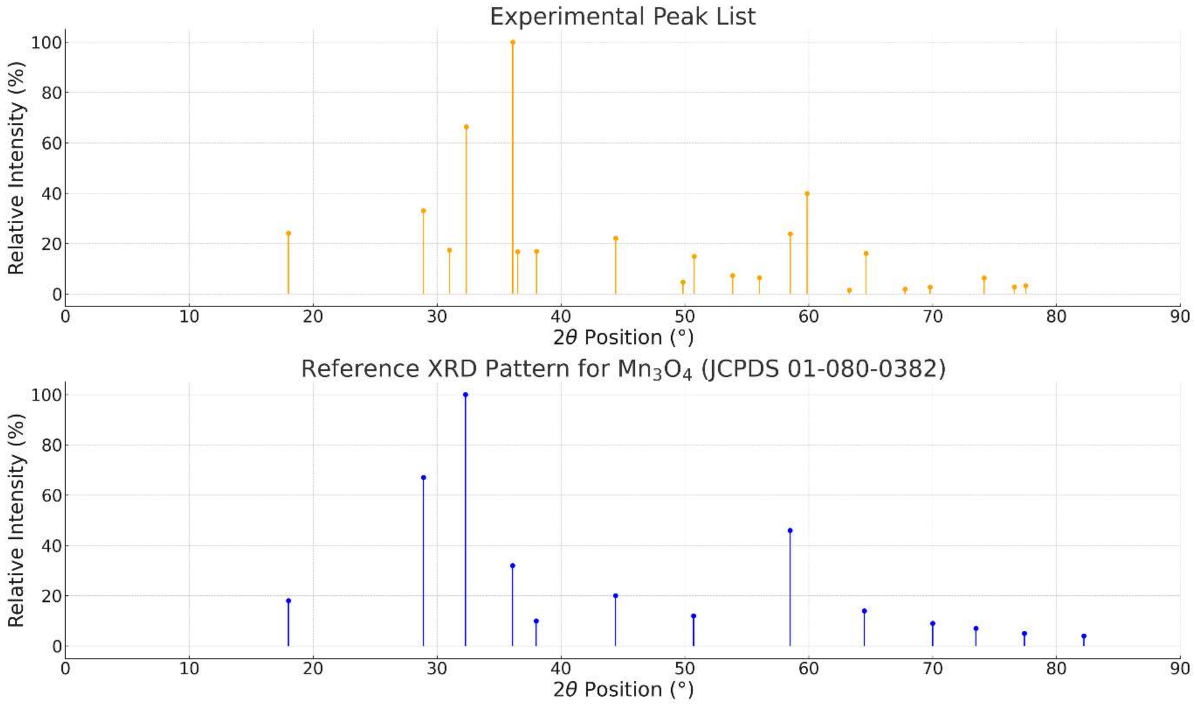

Figure S10. X-ray diffraction (XRD) pattern of the synthesized Mn_3O_4 nanoparticles (orange), overlaid with the standard reference pattern for Hausmannite Mn_3O_4 from the JCPDS database (card no. 01-080-0382) and the RRUFF database (card no. R040090, https://rruff.info/hausmannite/display=default/R040090) shown in blue. The key diffraction peaks are assigned to the tetragonal Mn_3O_4 phase, with corresponding Miller indices (hkl) listed in Table S4b. The reference pattern confirms the successful identification of Mn_3O_4 crystalline phases.

XPS. XPS analysis revealed the presence of the 3+ and 4+ oxides, achieved using prescribed peak fitting models for Mn_2O_3 and MnO_2. However, the distinction of Mn_3O_4, which exhibits both +2 and +3 oxidation states, is challenging due to similarities in peak shapes exhibited. [2] This discrepancy can be attributed to XPS's surface-sensitive nature, which samples only a limited region of the nanoparticles.

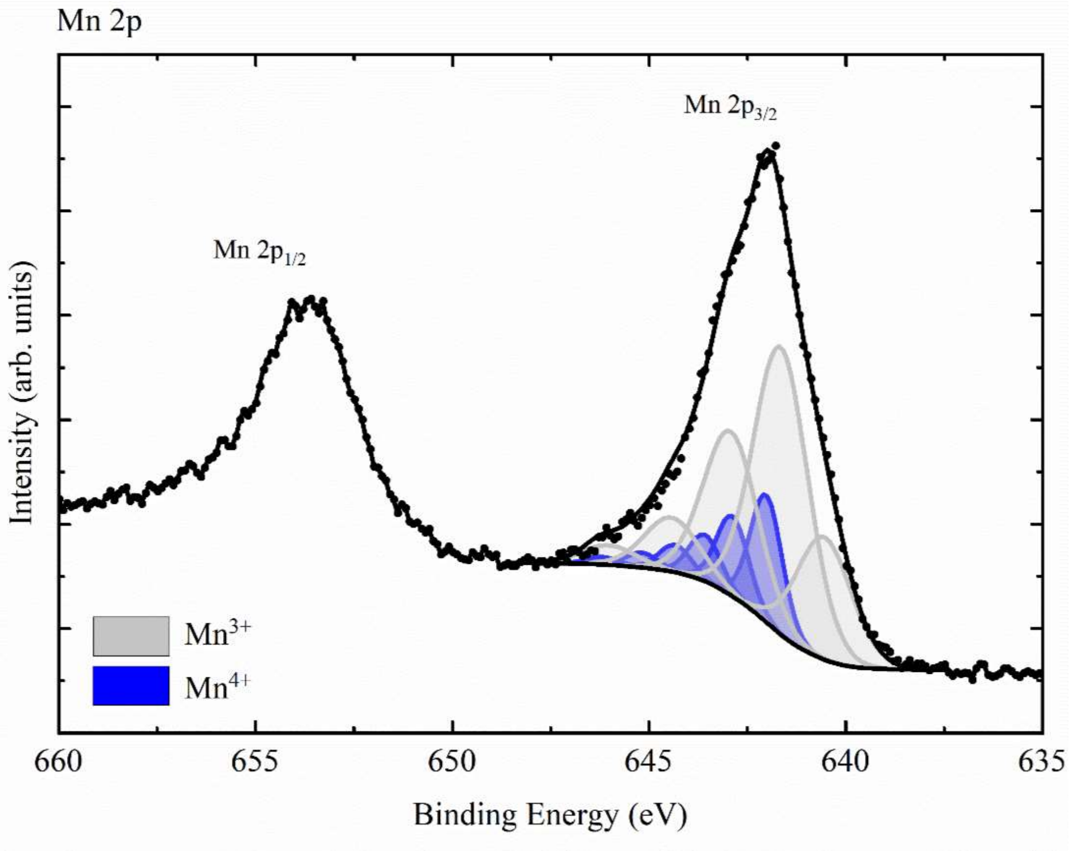

Figure S11. X-ray photoelectron spectra of the Mn3O4 nanoparticles showing the Mn 2p region, which is represented by the black line in the graph. The grey-colored peaks indicate the presence of the Mn3+ oxidation state, while the blue-colored peaks correspond to the Mn4+ oxidation state. These fitted peaks illustrate the contributions of different manganese oxidation states in the sample.

TEM. Figure S12 presents an electron micrograph illustrating the spherical morphology and good dispersion (i.e., lack of aggregation) of Mn_3O_4 nanoparticles and a histogram depicting their size distribution. The TEM image reveals that the nanoparticles are relatively uniform in shape, though some size variations are observed. Based on measurements from 100 particles across two sample regions, the size distribution analysis exhibits a near-normal distribution, with most particles centered around 50 nm. A slight tail in the distribution toward larger sizes suggests some polydispersity. The measured average diameter of 49.7 ± 10.3 nm indicates that the synthesis conditions led to relatively controlled nucleation and growth, with the moderate standard deviation (~10 nm) reflecting some variation in particle formation, likely due to differences in reaction kinetics or local concentration effects.

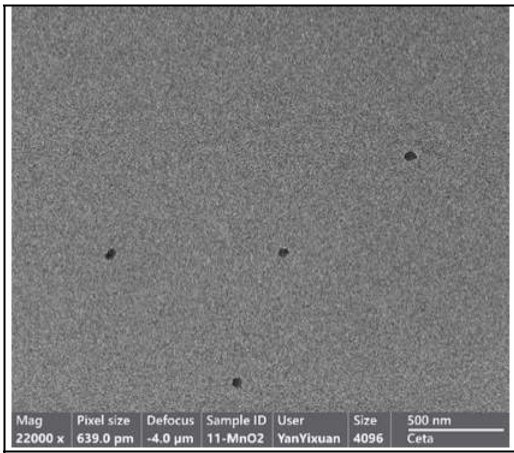

A

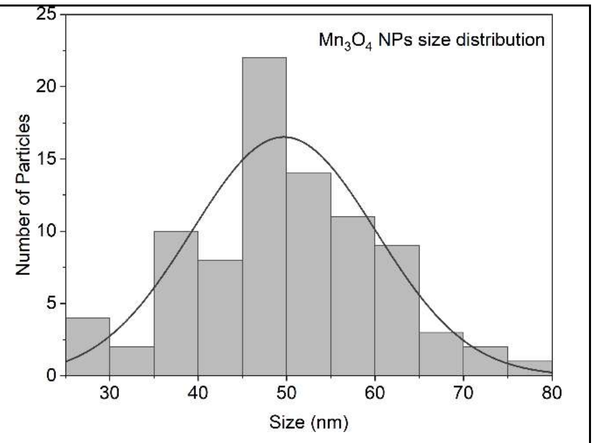

B

Figure S12. A) Transmission electron micrograph illustrating the size and morphology of the Mn_3O_4 nanoparticles and B) Size distribution analysis based on 100 particles from various regions across two separate samples, revealing an average diameter of d_{TEM}: 49.7 ± 10.3 nm.

DLS. The findings from Figure S10 complement the TEM and size distribution analysis from Figure S12, providing a more comprehensive characterization of the Mn_3O_4 nanoparticles. While the TEM analysis in Figure S9 showed a relatively uniform morphology with an average particle size of 49.7 ± 10.3 nm, the DLS measurements in Figure S10 indicate a slightly larger hydrodynamic diameter of 61.10 ± 14.05 nm. This difference can be attributed to the fact that DLS measures the hydrodynamic size, which includes the solvent layer surrounding the particles. In contrast, TEM provides a dry-state measurement of the core structure. The moderate polydispersity index (PDI) of 0.222 suggests that the nanoparticles maintain a relatively controlled size distribution in solution, aligning with the near-normal distribution observed in the TEM-derived histogram. Additionally, the zeta potential of +31.6 ± 7.84 mV confirms good colloidal stability, supporting the observation from TEM that the particles are well-dispersed with minimal aggregation. The slight tailing effect in the TEM size distribution also correlates with the PDI value, indicating that some larger particles or minor agglomeration may be present in the suspension. Together, these results validate the successful synthesis of Mn_3O_4 nanoparticles with controlled size, good dispersity, and stable colloidal behavior.

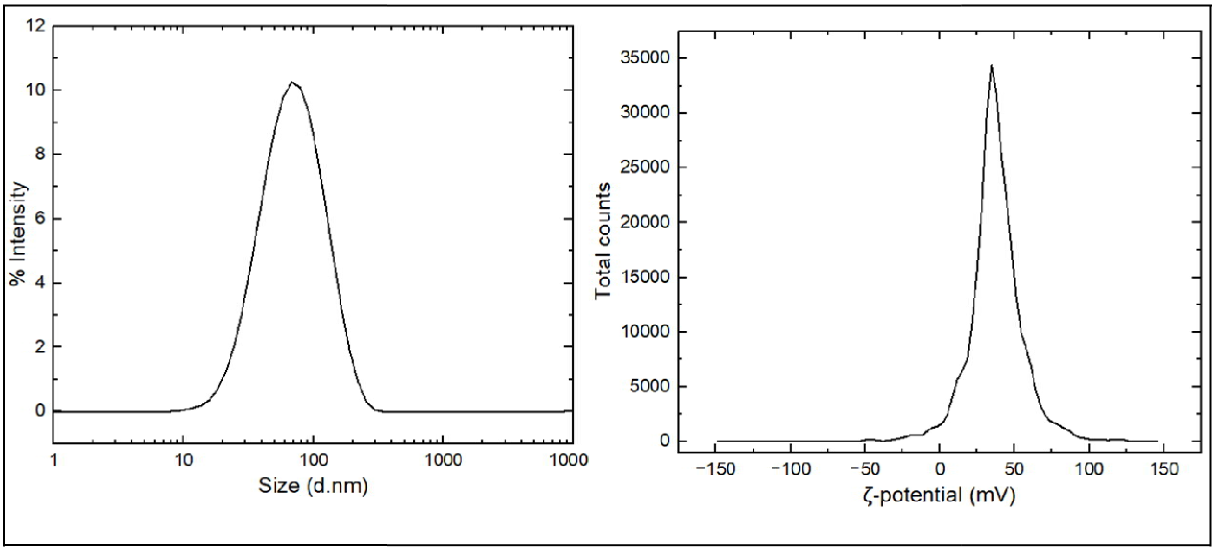

A	B

Figure S13. (A) Intensity-weighted size distribution of Mn_3O_4 nanoparticles measured using dynamic light scattering, showing a monomodal distribution with an average hydrodynamic diameter (d_h) of 61.10 ± 14.05 nm and a polydispersity index (PDI) of 0.222 ± 0.017. (B) Zeta potential distribution of the same particles with an average value of 31.6 ± 7.84 mV, indicating a positively charged surface and good colloidal stability.

1.7. Characterization of bifunctional organic-inorganic microreactors

Quantification of immobilized Mn_3O_4 nanoparticles. Immobilization of Mn_3O_4 was quantified spectroscopically. Absorbance at 385 nm of different Mn_3O_4 solutions with concentrations ranging from 5 µg mL^{-1} to 330 µg mL^{-1} was measured and plotted against the concentration to obtain a calibration curve. The calibration curve showed a good fitting in the range from 5 µg mL^{-1} to 330 µg mL^{-1}. The samples before and after the incubation with the $CaCO_3$ microparticles were diluted and the absorbance at 385 nm was subsequently measured. The concentration was calculated by interpolating the absorbance at 385 nm of the sample in the calibration curve.

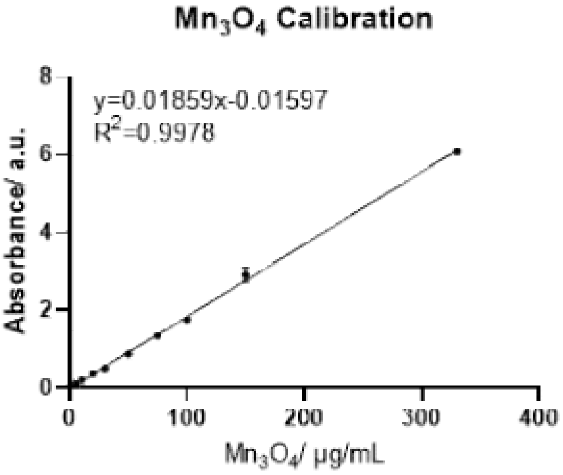

Figure S14. Calibration curve used to measure the Mn3O4 concentration in solution.

FTIR analysis of the S10 and S11 samples. Bare calcite exhibited three characteristic bands at 1409 cm^{-1}, 864 cm^{-1}, and 725 cm^{-1}, corresponding to the ν_3 asymmetric, ν_2 asymmetric, and ν_4 symmetric vibrations of the carbonate ion, respectively. [3] After the assembly of the microreactors these bands were conserved, and new ones are appeared.

A band at 1640 cm^{-1} was present in both microreactors, S10 and S11. The bands between 1250 cm^{-1} and 970 cm^{-1} can be assigned to different vibrations of the R-SO^{3-}. [4] These bands suggest the incorporation of PSS within the microreactors and correlate with the changes in ζ-potential during their assembly. Both microreactors exhibited a band at 1640 cm^{-1}, which can also be attributed to the presence of PSS and overlaps with the amide I band of proteins. In S11, a new peak emerged at 1475 cm^{-1}, overlapping with the ν_3 asymmetric band of the carbonate ion. This band can be attributed to the NH^+ δ confirming the incorporation of PAH during the assembly of the microreactors. Both of the microreactors exhibit bands around 3000 cm^{-1} attributed to the C-H stretches confirming the incorporation of organic material to the microreactors.

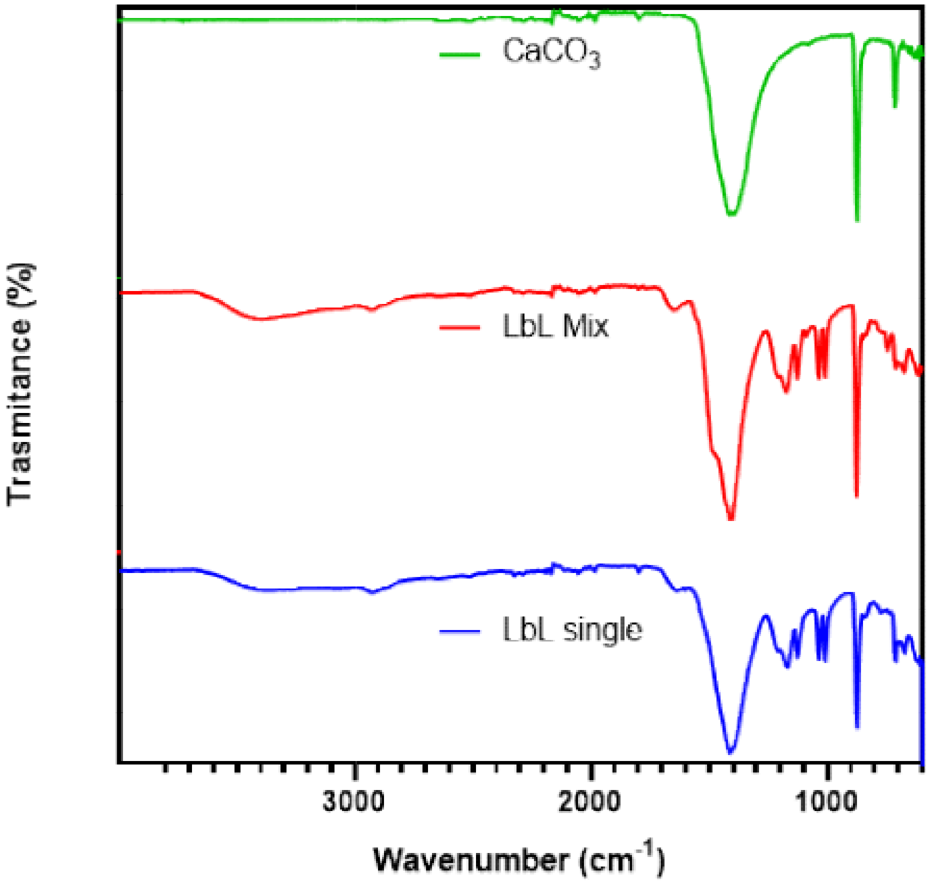

Figure S15. FTIR analysis of CaCO3 and S10 (LbL Mix) and S11 (LbL single) samples.

Quantification of immobilized GOx@APTAC. The immobilization of Gox@APTAC SENs was calculated with a Pierce BCA Assay kit from ThermoScientific.

Quantification of immobilized GOx@AA. For the quantification of the immobilized enzyme, UV absorption was measured. An aliquot of the solution was withdrawn before the enzyme immobilization and after immobilization. Enzyme concentration was assessed spectroscopically by measuring the absorption at 280 nm ($\varepsilon_{GOx(280nm)}$= 270000 M^{-1} cm^{-1}). Absorption of the different washings was also measured.

Glucose consumption. Microreactors (1 mg/mL, 1 mL) were incubated for 120 min with glucose (5 mM, 1 mL) in PBS (1 mL), aliquots were withdrawn at 15, 30 and 60 min to study the kinetics of glucose consumption. The glucose consumption by the microreactors was quantified by the DNS assay. For this, the DNS (10 g L^{-1}) was dissolved in MilliQ water together with phenol (2 g L^{-1}), sodium sulphite (0.5 g L^{-1}) and sodium hydroxide (10 g L^{-1}). This solution (100 µL) was added to the glucose sample (100 µL) and heated at 100 ºC for 30 min. Before measuring the absorbance at 575 nm the sample was cool down to room temperature. The concentration of the sample was quantified by interpolation of a calibration line ranging from 1 to 10 mM of glucose.

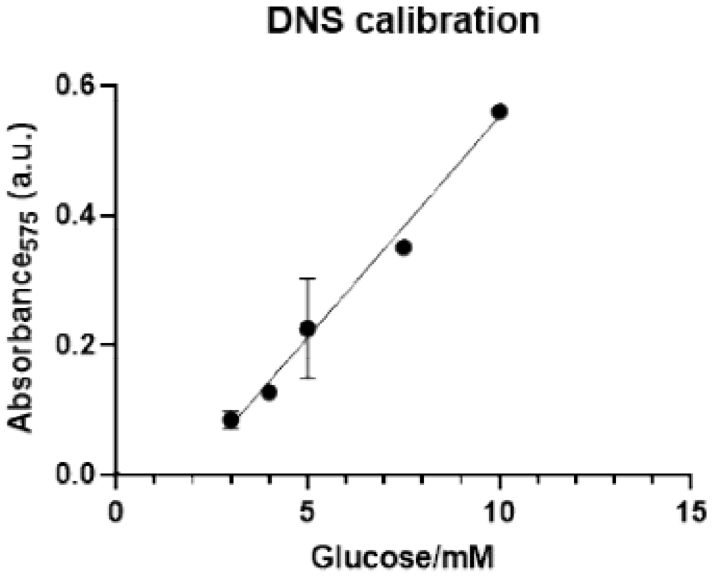

Figure S16. Calibration curve measured for DNS protocol

Catalase-like activity. The different microreactors (1 mg mL^{-1}) were incubated with H2O2 (50 µM) in sodium acetate buffer (12.5 mM pH 6.0) at room temperature while shaking. After 15 min, the remaining H2O2 was measured. This measurement is carried out by the peroxidation of ABTS by HRP. For this, the sample is incubated with ABTS (1 mM) and HRP for 10 min while shaking. After this, the oxidation of ABTS at 418 nm (ε418nm=36000 M-1 cm-1) was quantified.

Peroxidase activity. The peroxidative potential under cascade conditions was assessed by the peroxidation of Amplex Red (AR). For this, the formulated microreactors (1 mg mL^{-1}) were incubated for 30 min with glucose (50 mM) and AR (60 µM) in acetate buffer (20 mM, pH 6.0). To follow the progress of the reaction, aliquots were withdrawn at 10, 20 and 30 min and the fluorescence of the samples was checked ($\lambda_{ex.}$= 571 nm, $\lambda_{em.}$= 585 nm).

1.8. Statistical analysis of biological experiments.

The tables below display Tukey's post-hoc analysis outcomes, detailing the mean differences between groups and their 95% confidence intervals (CI). Statistical significance is indicated in the 'Below threshold?' column, where a 'Yes' denotes a significant difference, accompanied by corresponding summaries.

Table S5a. Results of Tukey's multiple comparisons test conducted on human pancreatic stellate cells (hPSCs) at 24h. This analysis was performed following a mixed-effects one-way analysis of variance (ANOVA) to assess the variations among different treatment groups. Each experimental condition was replicated three times to ensure the reliability of the results (* $p < 0.05$, ** $p < 0.01$, *** $p < 0.001$, **** $p < 0.0001$, ns: not significant).

Tukey's multiple comparisons test	Mean Diff.	95.00% CI of diff.	Below threshold?	Summary
CTRL vs. Vehicle	-0.6444	-17.24 to 15.95	No	ns

CTRL vs. LBL-Mn - 1	8.656	-50.02 to 67.33	No	ns
CTRL vs. LBL-Mn - 5	13.24	-2.039 to 28.53	No	ns
CTRL vs. LBL-Mn - 10	14.78	-2.562 to 32.12	No	ns
CTRL vs. LBL-GOx - 1	-7.800	-24.82 to 9.218	No	ns
CTRL vs. LBL-GOx - 5	1.400	-15.99 to 18.79	No	ns
CTRL vs. LBL-GOx - 10	17.46	-0.04121 to 34.95	No	ns
CTRL vs. LBL-GOx-Mn - 1	3.100	-8.630 to 14.83	No	ns
CTRL vs. LBL-GOx-Mn - 5	69.47	43.97 to 94.97	Yes	***
CTRL vs. LBL-GOx-Mn - 10	98.88	87.25 to 110.5	Yes	****
Vehicle vs. LBL-Mn - 1	9.300	-43.50 to 62.10	No	ns
Vehicle vs. LBL-Mn - 5	13.89	-5.145 to 32.92	No	ns
Vehicle vs. LBL-Mn - 10	15.42	0.2122 to 30.63	Yes	*
Vehicle vs. LBL-GOx - 1	-7.156	-20.93 to 6.623	No	ns
Vehicle vs. LBL-GOx - 5	2.044	-16.12 to 20.21	No	ns
Vehicle vs. LBL-GOx - 10	18.10	6.169 to 30.03	Yes	**
Vehicle vs. LBL-GOx-Mn - 1	3.744	-10.58 to 18.07	No	ns
Vehicle vs. LBL-GOx-Mn - 5	70.12	38.63 to 101.6	Yes	***
Vehicle vs. LBL-GOx-Mn - 10	99.52	86.44 to 112.6	Yes	****
LBL-Mn - 1 vs. LBL-Mn - 5	4.589	-52.23 to 61.41	No	ns
LBL-Mn - 1 vs. LBL-Mn - 10	6.122	-34.50 to 46.75	No	ns
LBL-Mn - 1 vs. LBL-GOx - 1	-16.46	-66.08 to 33.17	No	ns
LBL-Mn - 1 vs. LBL-GOx - 5	-7.256	-49.95 to 35.44	No	ns
LBL-Mn - 1 vs. LBL-GOx - 10	8.800	-40.06 to 57.66	No	ns
LBL-Mn - 1 vs. LBL-GOx-Mn - 1	-5.556	-53.88 to 42.76	No	ns
LBL-Mn - 1 vs. LBL-GOx-Mn - 5	60.82	10.02 to 111.6	Yes	*
LBL-Mn - 1 vs. LBL-GOx-Mn - 10	90.22	45.51 to 134.9	Yes	***
LBL-Mn - 5 vs. LBL-Mn - 10	1.533	-20.10 to 23.16	No	ns
LBL-Mn - 5 vs. LBL-GOx - 1	-21.04	-49.46 to 7.376	No	ns
LBL-Mn - 5 vs. LBL-GOx - 5	-11.84	-42.64 to 18.95	No	ns
LBL-Mn - 5 vs. LBL-GOx - 10	4.211	-20.79 to 29.21	No	ns
LBL-Mn - 5 vs. LBL-GOx-Mn - 1	-10.14	-28.04 to 7.753	No	ns
LBL-Mn - 5 vs. LBL-GOx-Mn - 5	56.23	35.54 to 76.91	Yes	***
LBL-Mn - 5 vs. LBL-GOx-Mn - 10	85.63	65.09 to 106.2	Yes	****
LBL-Mn - 10 vs. LBL-GOx - 1	-22.58	-39.05 to -6.105	Yes	**
LBL-Mn - 10 vs. LBL-GOx - 5	-13.38	-27.54 to 0.7890	No	ns
LBL-Mn - 10 vs. LBL-GOx - 10	2.678	-13.54 to 18.89	No	ns
LBL-Mn - 10 vs. LBL-GOx-Mn - 1	-11.68	-21.90 to -1.456	Yes	*
LBL-Mn - 10 vs. LBL-GOx-Mn - 5	54.69	26.56 to 82.83	Yes	**
LBL-Mn - 10 vs. LBL-GOx-Mn - 10	84.10	77.79 to 90.41	Yes	****
LBL-GOx - 1 vs. LBL-GOx - 5	9.200	-1.621 to 20.02	No	ns
LBL-GOx - 1 vs. LBL-GOx - 10	25.26	16.45 to 34.06	Yes	****
LBL-GOx - 1 vs. LBL-GOx-Mn - 1	10.90	-3.291 to 25.09	No	ns
LBL-GOx - 1 vs. LBL-GOx-Mn - 5	77.27	37.27 to 117.3	Yes	**
LBL-GOx - 1 vs. LBL-GOx-Mn - 10	106.7	94.09 to 119.3	Yes	****

LBL-GOx - 5 vs. LBL-GOx - 10	16.06	4.681 to 27.43	Yes	**
LBL-GOx - 5 vs. LBL-GOx-Mn - 1	1.700	-14.68 to 18.08	No	ns
LBL-GOx - 5 vs. LBL-GOx-Mn - 5	68.07	29.38 to 106.8	Yes	**
LBL-GOx - 5 vs. LBL-GOx-Mn - 10	97.48	85.66 to 109.3	Yes	****
LBL-GOx - 10 vs. LBL-GOx-Mn - 1	-14.36	-28.90 to 0.1850	No	ns
LBL-GOx - 10 vs. LBL-GOx-Mn - 5	52.02	15.52 to 88.51	Yes	**
LBL-GOx - 10 vs. LBL-GOx-Mn - 10	81.42	69.46 to 93.38	Yes	****
LBL-GOx-Mn - 1 vs. LBL-GOx-Mn - 5	66.37	40.48 to 92.26	Yes	***
LBL-GOx-Mn - 1 vs. LBL-GOx-Mn - 10	95.78	89.26 to 102.3	Yes	****
LBL-GOx-Mn - 5 vs. LBL-GOx-Mn - 10	29.41	2.550 to 56.26	Yes	*

Table S5b. Results of Tukey's multiple comparisons test conducted on human pancreatic stellate cells (hPSCs) at 72h. This analysis was performed following a repeated measurements one-way analysis of variance (ANOVA) to assess the variations among different treatment groups. Each experimental condition was replicated three times to ensure the reliability of the results (* $p < 0.05$, ** $p < 0.01$, *** $p < 0.001$, **** $p < 0.0001$, ns: not significant).

Tukey's multiple comparisons test	Mean Diff.	95.00% CI of diff.	Below threshold?	Summary
CTRL vs. Vehicle	-2.189	-14.19 to 9.811	No	ns
CTRL vs. LBL-Mn - 1	-1.211	-16.52 to 14.10	No	ns
CTRL vs. LBL-Mn - 5	-6.611	-21.83 to 8.605	No	ns
CTRL vs. LBL-Mn - 10	0.5556	-19.77 to 20.88	No	ns
CTRL vs. LBL-GOx - 1	-0.2333	-20.93 to 20.46	No	ns
CTRL vs. LBL-GOx - 5	3.033	-24.96 to 31.03	No	ns
CTRL vs. LBL-GOx - 10	3.722	-17.29 to 24.74	No	ns
CTRL vs. LBL-GOx-Mn - 1	3.400	-11.00 to 17.80	No	ns
CTRL vs. LBL-GOx-Mn - 5	84.94	71.43 to 98.45	Yes	****
CTRL vs. LBL-GOx-Mn - 10	99.04	92.49 to 105.6	Yes	****
Vehicle vs. LBL-Mn - 1	0.9778	-9.813 to 11.77	No	ns
Vehicle vs. LBL-Mn - 5	-4.422	-15.29 to 6.447	No	ns
Vehicle vs. LBL-Mn - 10	2.744	-13.54 to 19.03	No	ns
Vehicle vs. LBL-GOx - 1	1.956	-15.79 to 19.71	No	ns
Vehicle vs. LBL-GOx - 5	5.222	-18.94 to 29.39	No	ns
Vehicle vs. LBL-GOx - 10	5.911	-11.36 to 23.18	No	ns
Vehicle vs. LBL-GOx-Mn - 1	5.589	-9.382 to 20.56	No	ns
Vehicle vs. LBL-GOx-Mn - 5	87.13	67.98 to 106.3	Yes	****
Vehicle vs. LBL-GOx-Mn - 10	101.2	88.70 to 113.8	Yes	****
LBL-Mn - 1 vs. LBL-Mn - 5	-5.400	-10.57 to -0.2335	Yes	*
LBL-Mn - 1 vs. LBL-Mn - 10	1.767	-9.702 to 13.24	No	ns
LBL-Mn - 1 vs. LBL-GOx - 1	0.9778	-11.11 to 13.07	No	ns
LBL-Mn - 1 vs. LBL-GOx - 5	4.244	-11.55 to 20.04	No	ns
LBL-Mn - 1 vs. LBL-GOx - 10	4.933	-6.888 to 16.75	No	ns
LBL-Mn - 1 vs. LBL-GOx-Mn - 1	4.611	-4.035 to 13.26	No	ns
LBL-Mn - 1 vs. LBL-GOx-Mn - 5	86.16	63.88 to 108.4	Yes	****

LBL-Mn - 1 vs. LBL-GOx-Mn - 10	100.3	86.09 to 114.4	Yes	****
LBL-Mn - 5 vs. LBL-Mn - 10	7.167	-1.288 to 15.62	No	ns
LBL-Mn - 5 vs. LBL-GOx - 1	6.378	-4.162 to 16.92	No	ns
LBL-Mn - 5 vs. LBL-GOx - 5	9.644	-7.109 to 26.40	No	ns
LBL-Mn - 5 vs. LBL-GOx - 10	10.33	1.053 to 19.61	Yes	*
LBL-Mn - 5 vs. LBL-GOx-Mn - 1	10.01	2.900 to 17.12	Yes	**
LBL-Mn - 5 vs. LBL-GOx-Mn - 5	91.56	69.05 to 114.1	Yes	****
LBL-Mn - 5 vs. LBL-GOx-Mn - 10	105.7	91.45 to 119.9	Yes	****
LBL-Mn - 10 vs. LBL-GOx - 1	-0.7889	-10.12 to 8.538	No	ns
LBL-Mn - 10 vs. LBL-GOx - 5	2.478	-10.94 to 15.90	No	ns
LBL-Mn - 10 vs. LBL-GOx - 10	3.167	-4.282 to 10.62	No	ns
LBL-Mn - 10 vs. LBL-GOx-Mn - 1	2.844	-6.763 to 12.45	No	ns
LBL-Mn - 10 vs. LBL-GOx-Mn - 5	84.39	56.62 to 112.2	Yes	****
LBL-Mn - 10 vs. LBL-GOx-Mn - 10	98.49	79.06 to 117.9	Yes	****
LBL-GOx - 1 vs. LBL-GOx - 5	3.267	-10.69 to 17.22	No	ns
LBL-GOx - 1 vs. LBL-GOx - 10	3.956	-3.376 to 11.29	No	ns
LBL-GOx - 1 vs. LBL-GOx-Mn - 1	3.633	-5.564 to 12.83	No	ns
LBL-GOx - 1 vs. LBL-GOx-Mn - 5	85.18	56.18 to 114.2	Yes	****
LBL-GOx - 1 vs. LBL-GOx-Mn - 10	99.28	77.79 to 120.8	Yes	****
LBL-GOx - 5 vs. LBL-GOx - 10	0.6889	-14.48 to 15.86	No	ns
LBL-GOx - 5 vs. LBL-GOx-Mn - 1	0.3667	-16.20 to 16.93	No	ns
LBL-GOx - 5 vs. LBL-GOx-Mn - 5	81.91	46.34 to 117.5	Yes	***
LBL-GOx - 5 vs. LBL-GOx-Mn - 10	96.01	68.73 to 123.3	Yes	****
LBL-GOx - 10 vs. LBL-GOx-Mn - 1	-0.3222	-10.84 to 10.20	No	ns
LBL-GOx - 10 vs. LBL-GOx-Mn - 5	81.22	52.88 to 109.6	Yes	****
LBL-GOx - 10 vs. LBL-GOx-Mn - 10	95.32	74.41 to 116.2	Yes	****
LBL-GOx-Mn - 1 vs. LBL-GOx-Mn - 5	81.54	58.74 to 104.3	Yes	****
LBL-GOx-Mn - 1 vs. LBL-GOx-Mn - 10	95.64	81.44 to 109.8	Yes	****
LBL-GOx-Mn - 5 vs. LBL-GOx-Mn - 10	14.10	3.999 to 24.20	Yes	**

Table S6a. Results of Tukey's multiple comparisons test conducted on human pancreatic cancer cells (MiaPaCa-2) at 24h. This analysis was performed following a mixed-effects one-way analysis of variance (ANOVA) to assess the variations among different treatment groups. Each experimental condition was replicated three times to ensure the reliability of the results (* $p < 0.05$, ** $p < 0.01$, *** $p < 0.001$, **** $p < 0.0001$, ns: not significant).

**Tukey's multiple comparisons test**	**Mean Diff.**	**95.00% CI of diff.**	**Below threshold?**	**Summary**
CTRL vs. Vehicle	5.478	-21.35 to 32.31	No	ns
CTRL vs. LBL-Mn - 1	-1.433	-14.64 to 11.78	No	ns
CTRL vs. LBL-Mn - 5	1.467	-20.15 to 23.09	No	ns
CTRL vs. LBL-Mn - 10	1.144	-12.73 to 15.02	No	ns
CTRL vs. LBL-GOx - 1	-2.067	-21.07 to 16.93	No	ns
CTRL vs. LBL-GOx - 5	-2.056	-28.64 to 24.53	No	ns

CTRL vs. LBL-GOx - 10	-7.044	-42.64 to 28.55	No	ns
CTRL vs. LBL-GOx-Mn - 1	-17.50	-45.06 to 10.06	No	ns
CTRL vs. LBL-GOx-Mn - 5	14.09	-20.52 to 48.70	No	ns
CTRL vs. LBL-GOx-Mn - 10	72.62	34.89 to 110.3	Yes	**
Vehicle vs. LBL-Mn - 1	-6.911	-31.23 to 17.41	No	ns
Vehicle vs. LBL-Mn - 5	-4.011	-26.42 to 18.40	No	ns
Vehicle vs. LBL-Mn - 10	-4.333	-23.34 to 14.67	No	ns
Vehicle vs. LBL-GOx - 1	-7.544	-28.44 to 13.35	No	ns
Vehicle vs. LBL-GOx - 5	-7.533	-25.94 to 10.88	No	ns
Vehicle vs. LBL-GOx - 10	-12.52	-37.93 to 12.89	No	ns
Vehicle vs. LBL-GOx-Mn - 1	-22.98	-43.70 to -2.256	Yes	*
Vehicle vs. LBL-GOx-Mn - 5	8.611	-19.46 to 36.68	No	ns
Vehicle vs. LBL-GOx-Mn - 10	67.14	12.29 to 122.0	Yes	*
LBL-Mn - 1 vs. LBL-Mn - 5	2.900	-13.43 to 19.23	No	ns
LBL-Mn - 1 vs. LBL-Mn - 10	2.578	-13.01 to 18.17	No	ns
LBL-Mn - 1 vs. LBL-GOx - 1	-0.6333	-16.55 to 15.28	No	ns
LBL-Mn - 1 vs. LBL-GOx - 5	-0.6222	-19.15 to 17.91	No	ns
LBL-Mn - 1 vs. LBL-GOx - 10	-5.611	-34.13 to 22.90	No	ns
LBL-Mn - 1 vs. LBL-GOx-Mn - 1	-16.07	-38.38 to 6.247	No	ns
LBL-Mn - 1 vs. LBL-GOx-Mn - 5	15.52	-16.72 to 47.77	No	ns
LBL-Mn - 1 vs. LBL-GOx-Mn - 10	74.05	33.66 to 114.4	Yes	**
LBL-Mn - 5 vs. LBL-Mn - 10	-0.3222	-12.98 to 12.33	No	ns
LBL-Mn - 5 vs. LBL-GOx - 1	-3.533	-16.73 to 9.660	No	ns
LBL-Mn - 5 vs. LBL-GOx - 5	-3.522	-24.13 to 17.09	No	ns
LBL-Mn - 5 vs. LBL-GOx - 10	-8.511	-36.42 to 19.40	No	ns
LBL-Mn - 5 vs. LBL-GOx-Mn - 1	-18.97	-38.92 to 0.9833	No	ns
LBL-Mn - 5 vs. LBL-GOx-Mn - 5	12.62	-4.661 to 29.91	No	ns
LBL-Mn - 5 vs. LBL-GOx-Mn - 10	71.15	29.90 to 112.4	Yes	**
LBL-Mn - 10 vs. LBL-GOx - 1	-3.211	-14.49 to 8.072	No	ns
LBL-Mn - 10 vs. LBL-GOx - 5	-3.200	-27.19 to 20.79	No	ns
LBL-Mn - 10 vs. LBL-GOx - 10	-8.189	-40.95 to 24.58	No	ns
LBL-Mn - 10 vs. LBL-GOx-Mn - 1	-18.64	-43.17 to 5.882	No	ns
LBL-Mn - 10 vs. LBL-GOx-Mn - 5	12.94	-7.009 to 32.90	No	ns
LBL-Mn - 10 vs. LBL-GOx-Mn - 10	71.47	32.45 to 110.5	Yes	**
LBL-GOx - 1 vs. LBL-GOx - 5	0.0111	-21.74 to 21.76	No	ns
LBL-GOx - 1 vs. LBL-GOx - 10	-4.978	-38.88 to 28.93	No	ns
LBL-GOx - 1 vs. LBL-GOx-Mn - 1	-15.43	-41.22 to 10.35	No	ns
LBL-GOx - 1 vs. LBL-GOx-Mn - 5	16.16	-4.943 to 37.25	No	ns
LBL-GOx - 1 vs. LBL-GOx-Mn - 10	74.68	33.87 to 115.5	Yes	**
LBL-GOx - 5 vs. LBL-GOx - 10	-4.989	-19.97 to 9.988	No	ns
LBL-GOx - 5 vs. LBL-GOx-Mn - 1	-15.44	-28.58 to -2.306	Yes	*
LBL-GOx - 5 vs. LBL-GOx-Mn - 5	16.14	-17.75 to 50.03	No	ns
LBL-GOx - 5 vs. LBL-GOx-Mn - 10	74.67	24.38 to 125.0	Yes	**
LBL-GOx - 10 vs. LBL-GOx-Mn - 1	-10.46	-23.85 to 2.942	No	ns

LBL-GOx - 10 vs. LBL-GOx-Mn - 5	21.13	-18.12 to 60.39	No	ns
LBL-GOx - 10 vs. LBL-GOx-Mn - 10	79.66	20.35 to 139.0	Yes	*
LBL-GOx-Mn - 1 vs. LBL-GOx-Mn - 5	31.59	2.716 to 60.46	Yes	*
LBL-GOx-Mn - 1 vs. LBL-GOx-Mn - 10	90.12	34.49 to 145.7	Yes	**
LBL-GOx-Mn - 5 vs. LBL-GOx-Mn - 10	58.53	-5.506 to 122.6	No	ns

Table S6b. Results of Tukey's multiple comparisons test conducted on human pancreatic cancer cells (MiaPaCa-2) at 72h. This analysis was performed following a repeated measurements one-way analysis of variance (ANOVA) to assess the variations among different treatment groups. Each experimental condition was replicated three times to ensure the reliability of the results. (* $p < 0.05$, ** $p < 0.01$, *** $p < 0.001$, **** $p < 0.0001$, ns: not significant.

**Tukey's multiple comparisons test**	**Mean Diff.**	**95.00% CI of diff.**	**Below threshold?**	**Summary**
CTRL vs. Vehicle	-5.656	-15.12 to 3.806	No	ns
CTRL vs. LBL-Mn - 1	-0.3889	-11.53 to 10.75	No	ns
CTRL vs. LBL-Mn - 5	-8.667	-22.39 to 5.060	No	ns
CTRL vs. LBL-Mn - 10	-2.967	-18.63 to 12.70	No	ns
CTRL vs. LBL-GOx - 1	-7.533	-20.76 to 5.690	No	ns
CTRL vs. LBL-GOx - 5	-7.422	-20.99 to 6.147	No	ns
CTRL vs. LBL-GOx - 10	-13.18	-28.84 to 2.481	No	ns
CTRL vs. LBL-GOx-Mn - 1	-6.644	-17.57 to 4.283	No	ns
CTRL vs. LBL-GOx-Mn - 5	20.53	3.806 to 37.26	Yes	*
CTRL vs. LBL-GOx-Mn - 10	77.83	47.43 to 108.2	Yes	***
Vehicle vs. LBL-Mn - 1	5.267	-3.946 to 14.48	No	ns
Vehicle vs. LBL-Mn - 5	-3.011	-12.76 to 6.741	No	ns
Vehicle vs. LBL-Mn - 10	2.689	-13.25 to 18.62	No	ns
Vehicle vs. LBL-GOx - 1	-1.878	-14.87 to 11.12	No	ns
Vehicle vs. LBL-GOx - 5	-1.767	-11.55 to 8.012	No	ns
Vehicle vs. LBL-GOx - 10	-7.522	-21.90 to 6.858	No	ns
Vehicle vs. LBL-GOx-Mn - 1	-0.9889	-13.31 to 11.33	No	ns
Vehicle vs. LBL-GOx-Mn - 5	26.19	8.452 to 43.93	Yes	**
Vehicle vs. LBL-GOx-Mn - 10	83.49	53.88 to 113.1	Yes	****
LBL-Mn - 1 vs. LBL-Mn - 5	-8.278	-20.96 to 4.409	No	ns
LBL-Mn - 1 vs. LBL-Mn - 10	-2.578	-11.92 to 6.769	No	ns
LBL-Mn - 1 vs. LBL-GOx - 1	-7.144	-19.80 to 5.515	No	ns
LBL-Mn - 1 vs. LBL-GOx - 5	-7.033	-14.15 to 0.08700	No	ns
LBL-Mn - 1 vs. LBL-GOx - 10	-12.79	-28.08 to 2.506	No	ns
LBL-Mn - 1 vs. LBL-GOx-Mn - 1	-6.256	-15.75 to 3.239	No	ns
LBL-Mn - 1 vs. LBL-GOx-Mn - 5	20.92	5.605 to 36.24	Yes	**
LBL-Mn - 1 vs. LBL-GOx-Mn - 10	78.22	53.05 to 103.4	Yes	****
LBL-Mn - 5 vs. LBL-Mn - 10	5.700	-8.355 to 19.75	No	ns
LBL-Mn - 5 vs. LBL-GOx - 1	1.133	-7.667 to 9.933	No	ns
LBL-Mn - 5 vs. LBL-GOx - 5	1.244	-11.51 to 14.00	No	ns
LBL-Mn - 5 vs. LBL-GOx - 10	-4.511	-12.33 to 3.309	No	ns

LBL-Mn - 5 vs. LBL-GOx-Mn - 1	2.022	-9.355 to 13.40	No	ns
LBL-Mn - 5 vs. LBL-GOx-Mn - 5	29.20	14.16 to 44.24	Yes	***
LBL-Mn - 5 vs. LBL-GOx-Mn - 10	86.50	58.79 to 114.2	Yes	****
LBL-Mn - 10 vs. LBL-GOx - 1	-4.567	-16.58 to 7.447	No	ns
LBL-Mn - 10 vs. LBL-GOx - 5	-4.456	-18.97 to 10.06	No	ns
LBL-Mn - 10 vs. LBL-GOx - 10	-10.21	-23.86 to 3.433	No	ns
LBL-Mn - 10 vs. LBL-GOx-Mn - 1	-3.678	-13.00 to 5.646	No	ns
LBL-Mn - 10 vs. LBL-GOx-Mn - 5	23.50	8.861 to 38.14	Yes	**
LBL-Mn - 10 vs. LBL-GOx-Mn - 10	80.80	55.62 to 106.0	Yes	****
LBL-GOx - 1 vs. LBL-GOx - 5	0.1111	-14.30 to 14.52	No	ns
LBL-GOx - 1 vs. LBL-GOx - 10	-5.644	-11.92 to 0.6322	No	ns
LBL-GOx - 1 vs. LBL-GOx-Mn - 1	0.8889	-6.800 to 8.578	No	ns
LBL-GOx - 1 vs. LBL-GOx-Mn - 5	28.07	13.82 to 42.31	Yes	***
LBL-GOx - 1 vs. LBL-GOx-Mn - 10	85.37	62.09 to 108.6	Yes	****
LBL-GOx - 5 vs. LBL-GOx - 10	-5.756	-22.06 to 10.55	No	ns
LBL-GOx - 5 vs. LBL-GOx-Mn - 1	0.7778	-11.94 to 13.50	No	ns
LBL-GOx - 5 vs. LBL-GOx-Mn - 5	27.96	12.82 to 43.09	Yes	**
LBL-GOx - 5 vs. LBL-GOx-Mn - 10	85.26	62.44 to 108.1	Yes	****
LBL-GOx - 10 vs. LBL-GOx-Mn - 1	6.533	-3.621 to 16.69	No	ns
LBL-GOx - 10 vs. LBL-GOx-Mn - 5	33.71	17.42 to 50.00	Yes	***
LBL-GOx - 10 vs. LBL-GOx-Mn - 10	91.01	65.25 to 116.8	Yes	****
LBL-GOx-Mn - 1 vs. LBL-GOx-Mn - 5	27.18	12.44 to 41.92	Yes	**
LBL-GOx-Mn - 1 vs. LBL-GOx-Mn - 10	84.48	60.84 to 108.1	Yes	****
LBL-GOx-Mn - 5 vs. LBL-GOx-Mn - 10	57.30	35.25 to 79.35	Yes	****

REFERENCES

1. M. M. Bhuyan; M. Jophous; J.-H. Jeong, Synthesis and characterization of gamma radiation-induced (3-acrylamidopropyl) trimethylammonium chloride-acrylic acid functional superabsorbent hydrogel, *Polymer Bulletin.* 2022, **80**, 8651.
2. M. C. Biesinger; B. P. Payne; A. P. Grosvenor; L. W. M. Lau; A. R. Gerson; R. S. C. Smart, Resolving surface chemical states in XPS analysis of first row transition metals, oxides and hydroxides: Cr, Mn, Fe, Co and Ni, *App. Surf Sci.* 2011, **257**, 2717.
3. J. D. Rodriguez-Blanco; S. Shaw; L. G. Benning, The kinetics and mechanisms of amorphous calcium carbonate (ACC) crystallization to calcite, via vaterite, *Nanoscale.* 2011, **3**, 265.
4. Y. Wang; Y. Shen; Y. Zhang; B. Yue; C. Wu, pH-Sensitive Polyacrylic Acid (PAA) Hydrogels Trapped with Polysodium-p-Styrenesulfonate (PSS), *J. Macromol. Sci. Part B.* 2006, **45**, 563.
